# Light-induced gene expression with photocaged IPTG for induction profiling in a high-throughput screening system

**DOI:** 10.1186/s12934-016-0461-3

**Published:** 2016-04-23

**Authors:** Georg Wandrey, Claus Bier, Dennis Binder, Kyra Hoffmann, Karl-Erich Jaeger, Jörg Pietruszka, Thomas Drepper, Jochen Büchs

**Affiliations:** AVT-Biochemical Engineering, RWTH Aachen University, Worringerweg 1, Aachen, 52074 Germany; Institute of Bioorganic Chemistry, Heinrich-Heine-University Düsseldorf, Forschungszentrum Jülich, Jülich, 52426 Germany; Institute of Molecular Enzyme Technology, Heinrich-Heine-University Düsseldorf, Forschungszentrum Jülich, Jülich, 52426 Germany; Institut für Bio- und Geowissenschaften (IBG-1: Biotechnologie), Forschungszentrum Jülich, Jülich, 52428 Germany

**Keywords:** Caged compounds, Photocaged IPTG, LED array, Optical induction, Automatization, High-throughput screening, Recombinant protein expression, *Escherichia coli*, FbFP, Induction profiling, BioLector, Shaken microtiter plate

## Abstract

**Background:**

Inducible expression systems are frequently used for the production of heterologous proteins. Achieving maximum product concentrations requires induction profiling, namely the optimization of induction time and inducer concentration. However, the respective experiments can be very laborious and time-consuming. In this work, a new approach for induction profiling is presented where induction in a microtiter plate based cultivation system (BioLector) is achieved by light using photocaged isopropyl *β*-d-1-thiogalactopyranoside (cIPTG).

**Results:**

A flavin mononucleotide-based fluorescent reporter protein (FbFP) was expressed using a T7-RNA-polymerase dependent *E. coli* expression system which required IPTG as inducer. High power UV-A irradiation was directed into a microtiter plate by light-emitting diodes placed above each well of a 48-well plate. Upon UV irradiation, IPTG is released (uncaged) and induces product formation. IPTG uncaging, formation of the fluorescent reporter protein and biomass growth were monitored simultaneously in up to four 48-well microtiter plates in parallel with an in-house constructed BioLector screening system. The amount of released IPTG can be gradually and individually controlled for each well by duration of UV-A exposure, irradiance and concentration of photocaged IPTG added at the start of the cultivation. A comparison of experiments with either optical or conventional IPTG induction shows that product formation and growth are equivalent. Detailed induction profiles revealed that for the strain and conditions used maximum product formation is reached for very early induction times and with just 6–8 s of UV-A irradiation or 60–80 µM IPTG.

**Conclusions:**

Optical induction and online monitoring were successfully combined in a high-throughput screening system and the effect of optical induction with photocaged IPTG was shown to be equivalent to conventional induction with IPTG. In contrast to conventional induction, optical induction is less costly to parallelize, easy to automate, non-invasive and without risk of contamination. Therefore, light-induced gene expression with photocaged IPTG is a highly advantageous method for the efficient optimization of heterologous protein production and has the potential to replace conventional induction with IPTG.

**Electronic supplementary material:**

The online version of this article (doi:10.1186/s12934-016-0461-3) contains supplementary material, which is available to authorized users.

## Background

High productivity in heterologous protein production is achieved by optimization of strains, culture conditions and process related parameters. Inducible expression systems are applied to separate the cultivation into an initial growth phase for unimpeded biomass formation and a subsequent production phase where growth is impeded while metabolic resources are shifted towards product formation. The switch from growth to production phase (induction of target gene expression) can be achieved by addition of small chemical inducer molecules among which isopropyl β-d-1-thiogalactopyranoside (IPTG) is the most popular choice and widely applied, especially in lab scale. The amount of IPTG and the time when it is added during cultivation are crucial parameters for process performance [1, 2]. If induction is performed too early, biomass concentration is insufficient for reasonable protein production; if it is performed too late, not enough substrate is left for product formation. If IPTG concentration is not sufficiently high, cells may not reach their full expression potential; however, if IPTG concentration exceeds a critical limit, a balanced metabolism cannot be maintained and toxic effects might be observed [3–5].

The induction optimum depends on the respective strain, expression plasmid and target gene [2, 6]. Although general recommendations (i.e. ‘induce with 1000 µM IPTG at OD 0.6-1.2′ [1, 2]) commonly yield decent results and mathematical models for induction parameters have been described [5, 7, 8], the optimum parameters for each process still have to be verified in time-consuming experiments. Hence, microtiter plate based screening systems that allow parallelized and cost-effective experiments in small scale have successfully been applied [9, 10]. However, the addition of IPTG solution to small scale cultures is associated with some drawbacks which are usually not discussed in literature. For induction, either cumbersome manual intervention at each time of induction or cost-intensive investment into an automatized liquid handling system is required. Before addition of IPTG, the microtiter plate shaking is usually stopped which can result in an oxygen limitation [9]. Then, the sterile barrier on top of the microtiter plate is pierced or removed which necessitates precautions to avoid contamination [10]. The addition of an IPTG containing solution dilutes the cultures and additional pipetting is required if different amounts of IPTG have to be added to different wells but the dilution effect is to be kept constant. If the sterile barrier is pierced for pipetting, the barrier might not seal the well completely anymore once the pipettes or syringes are retracted. This can result in increased evaporation which affects results. If the sterile barrier is instead removed for pipetting, it has to be replaced either manually or by additional automated equipment, once a pipetting step is completed.

This complex procedure of adding IPTG solution is especially problematic when different clones of a clone library are to be compared regarding heterologous protein production. The clones should not be induced at the same time but when they reach the same optical density. Each time a single clone reaches the induction criterion the invasive induction procedure is activated which interrupts the measurement and affects the growth of all clones [9]. With ever increasing numbers of parallel cultivations individual induction with IPTG solution reaches its limits even with automated pipetting systems. Instead, a parallelized and less invasive induction method for small scale cultivations is highly desirable.

Non-invasive optical measuring techniques have already been applied extensively for online monitoring [11–13]. In this study, it was investigated whether non-invasive optical induction could be practical as well [14]. In 2007, Young and Deiters attached the photo-removable group 6-nitropiperonal to IPTG yielding a photocaged IPTG derivative (cIPTG) which serves as a dormant IPTG reservoir [15]. As long as the photocage is attached, cIPTG cannot bind to the *lac* repressor and no target gene is expressed. Upon UV-A irradiation IPTG is uncaged and can act as an inducer. Based on this concept, a device for individual optical induction of each well in a microtiter plate was constructed. A high-throughput screening system was then used for detailed induction profiling and to test whether optical induction with cIPTG could replace conventional induction with IPTG.

## Results and discussion

### LED array for optical induction

**Fig. 1:**
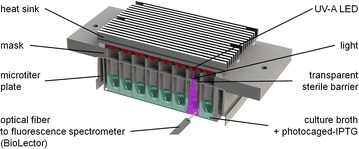
LED array for individual illumination of 48 wells in a microtiter plate. High performance UV-A LEDs are mounted onto a heat sink. Emitted light passes a transparent gas-permeable sterile barrier and reaches the subjacent well containing the culture and photocaged-IPTG (cIPTG). A mask ensures that no stray light can enter adjacent wells. IPTG is uncaged and induces formation of a fluorescent reporter protein. Protein and biomass concentrations are monitored through the transparent bottom of the microtiter plate using an in-house constructed BioLector setup. A robotic arm moves an optical fiber which is connected to a fluorescence spectrometer from well to well in rapid succession to allow quasi-continuous and non-invasive measurements during continuous orbital shaking for cultivation

To achieve optical induction, UV-A irradiation has to be introduced into the culture broth containing cIPTG to release uncaged IPTG from its photocage. We constructed an LED array with 48 UV-A LEDs (λ_max_ = 368 nm, Fig. 1). A high-performance UV-A LED is positioned directly above each corresponding well. Once an LED is switched on, UV-A irradiation passes the transparent sterile barrier on top of the microtiter plate and reaches the culture. There, the intense UV-A irradiation (52 mW/cm^2^) leads to cIPTG uncaging and subsequently expression of target genes is induced. A mask positioned between LEDs and microtiter plate ensures that per LED only one well is illuminated. Cross-illumination through the walls of neighbouring wells can be excluded because plates with black walls are used. The LED array can quickly be mounted on top of the microtiter plate where it is positioned by a notch and fixed by two screws. Due to its robust design the LED array can be used for illumination during cultivation under typical shaking conditions which are required for sufficient oxygen transfer and mixing [16].

### Online monitoring with BioLector screening system

The space below the microtiter plate is not affected by the illumination from above. Therefore, non-invasive online fluorescence measurements according to the BioLector design [11, 17] were performed through the transparent bottom using an in-house constructed BioLector prototype. A fluorescence excitation wavelength is chosen from the white spectrum of a xenon lamp by an excitation monochromator inside the fluorescence spectrometer and the light is guided to the microtiter plate through one branch of a Y-shaped optical fibre bundle. Through the other branch the resulting scattered light and fluorescence emission is guided back to the spectrometer where the light passes the emission monochromator and reaches the detector. The optical fibre bundle is moved by a robotic arm which allows measuring each culture in rapid succession without stopping the shaking movement of the well plate. Up to four microtiter plates in parallel can be monitored with an acquisition rate of one to two data points per second. In the most basic setup this device is used to gather quasi-continuous data on biomass growth and fluorescent protein formation by monitoring suitable excitation/emission wavelength combinations.

**Fig. 2:**
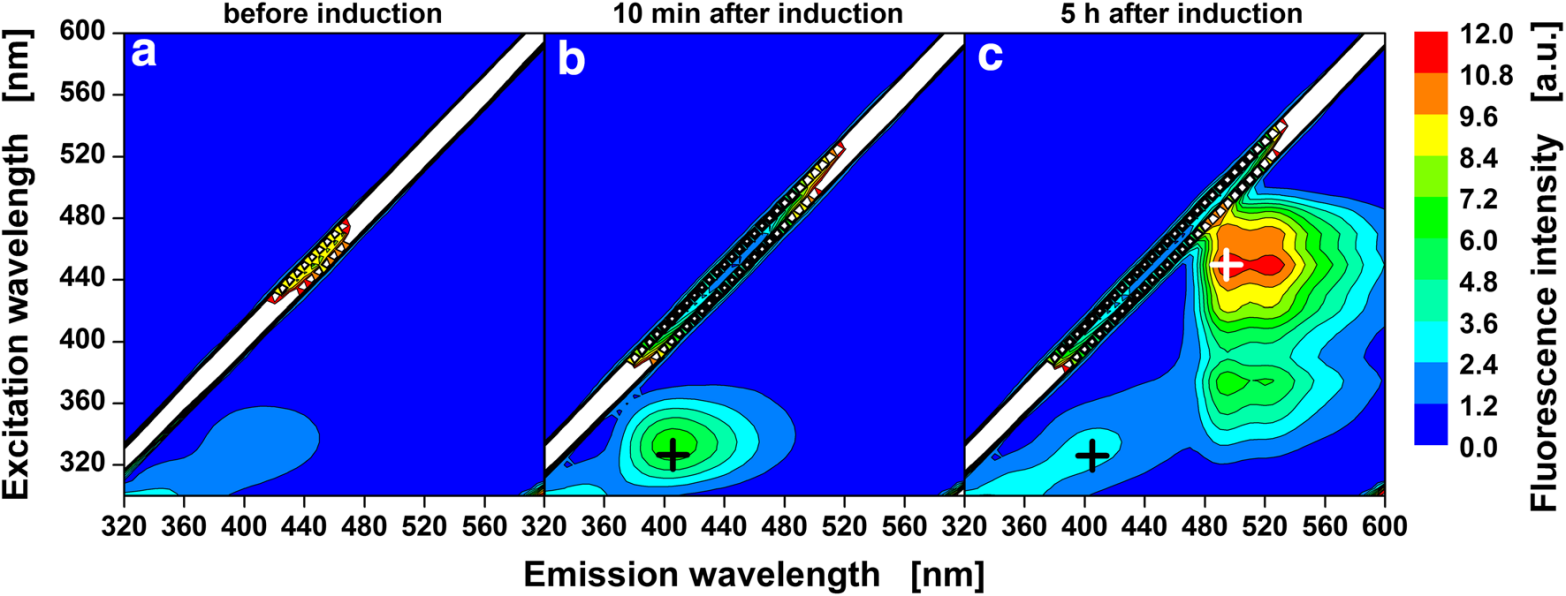
Fluorescence spectrum of an *E. coli* Tuner(DE3)/pRhotHi-2-LacI-EcFbFP culture before and after optical induction. 2D fluorescence scan with excitation wavelengths from 300–600 nm and emission wavelengths from 320–600 nm before (**a**) and after optical induction (**b**: 10 min, **c**: 5 h). Ten minutes after UV-A irradiation for optical induction a fluorescence signal with an excitation maximum at 335 nm and an emission maximum at 405 nm is detected (**b**). Five hours later this signal is reduced by about 60 % and a strong fluorescence signal of the target protein FbFP with an excitation maximum at 450 nm and an emission maximum at 495 nm is detected (**c**). Biomass autofluorescence was detected but is not visible in this plot because other fluorescence signals are much stronger. Cultivation conditions: 800 µL Wilms-MOPS mineral medium (20 g/L glucose, 0.2 M MOPS) per well in a 48-FlowerPlate, 30 °C, shaking frequency: 1000 rpm, shaking diameter: 3 mm, 400 µM cIPTG added at the start of cultivation, optical induction with LED array after 10 h of cell cultivation for 6 s (λ_max_ = 368 nm, I = 52 mW/cm^2^)

Suitable excitation/emission wavelength combinations for online monitoring can either be taken from literature or can also be extracted from 2D fluorescence scans obtained using the method described by Siepert et al. [18]. Fig. 2 shows 2D fluorescence spectra obtained at three time points during a cultivation of *E. coli* Tuner(DE3)/pRhotHi-2-LacI-EcFbFP. This strain was chosen for optical induction experiments in this work because it was already shown that the T7-RNA polymerase expression host *E. coli* Tuner(DE3) is well-suited for tight and gradual regulation of homogenous gene expression in combination with cIPTG [19]. At the beginning of the cultivation, cIPTG was added to a concentration of 400 µM. The first 2D fluorescence spectrum obtained right before optical induction (Fig. 2a) shows no fluorescence of the flavin mononucleotide-based fluorescent reporter protein (FbFP). Then, expression of FbFP reporter gene was induced by uncaging of cIPTG with UV-A irradiation using the LED array. The 2D fluorescence spectrum obtained 5 h after optical induction (Fig. 2c) shows a strong fluorescence signal with the spectral characteristics of FbFP [20]. This demonstrates successful optical induction with cIPTG and the constructed UV-A LED array. As reported previously, FbFP formation can be monitored at an excitation wavelength of 450 nm and an emission wavelength of 495 nm (Fig. 2c, white cross) [20].

### Identification of additional fluorescence signal

**Fig. 3:**
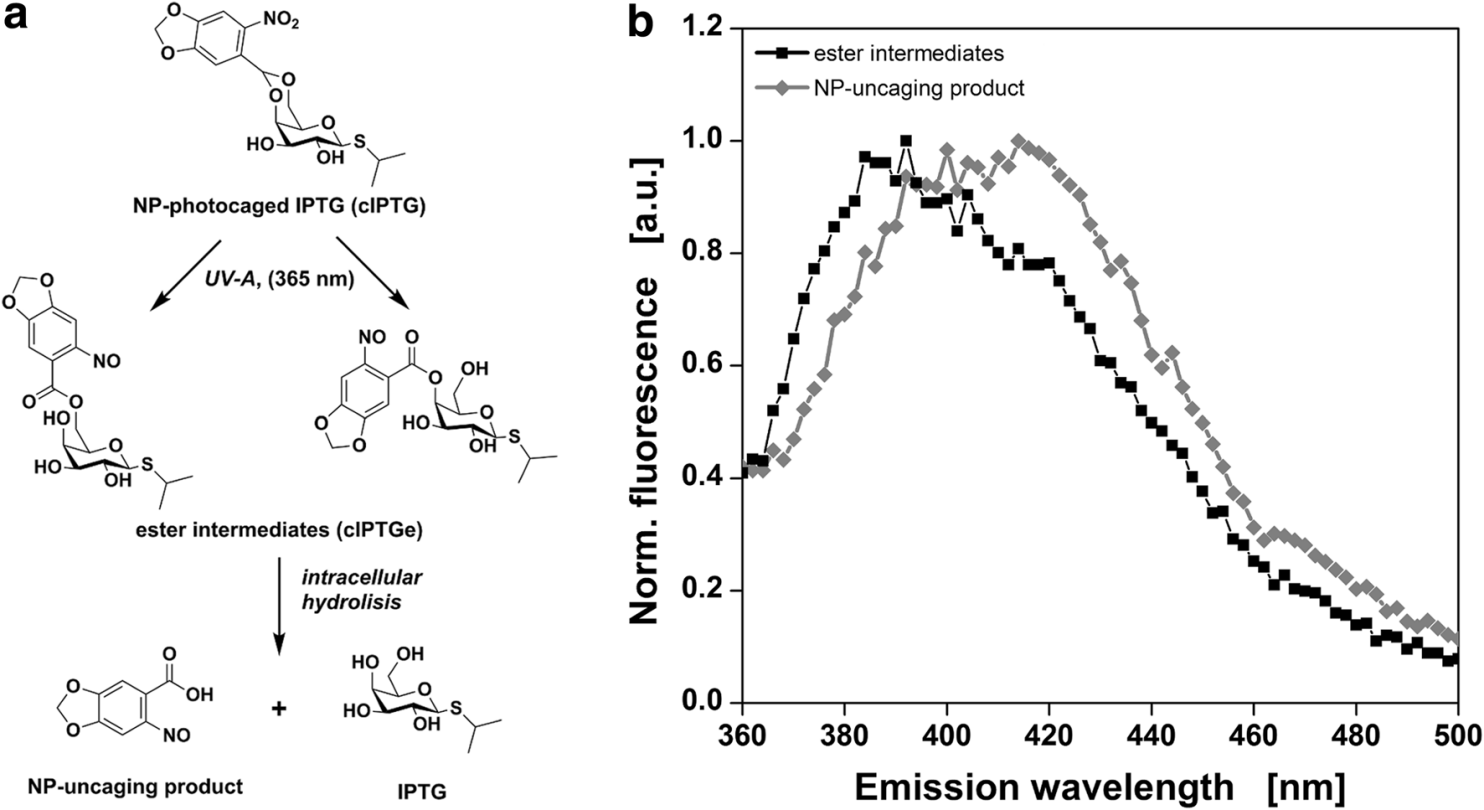
cIPTG uncaging mechanism and fluorescence emission spectra of caged IPTG ester intermediates and NP-uncaging product. The two-step cIPTG uncaging mechanism as described by Young and Deiters [15] **a** and normalized fluorescence emission spectra (λ_ex_ = 330 nm) of cIPTG ester intermediates isolated via HPLC (*black*) and nitropiperonal uncaging product (*grey*) in H_2_O (**b**)

Besides the FbFP signal an additional fluorescence signal (λ_ex,max_ = 335 nm, λ_em,max_ = 405 nm) appeared in the 2D fluorescence spectrum measured ten minutes after UV-A irradiation for optical induction (Fig. 2b). Five hours after UV-A irradiation (Fig. 2c) this signal is still visible but its intensity is lowered by about 60 %. Due to the appearance of this signal immediately after UV-A irradiation and its later decay, it was suspected that the signal might result from caged IPTG ester intermediates (cIPTGe, Fig. 3a) which are the photoproducts of cIPTG as described by Young and Deiters [15]. According to these authors, the ester intermediates are hydrolysed by intracellular esterases to yield the nitropiperonal uncaging product and free IPTG.

The fluorescence emission spectrum of caged IPTG ester intermediates that were isolated by HPLC and identified by NMR (Additional files 1, 2) are shown in Fig. 3b. The normalized emission spectrum with maximum emission at 417 nm (λ_ex_ = 330 nm) fits in well with the emission spectrum observed in vivo after photo-cleavage. Besides the ester intermediates the isolated nitropiperonal uncaging product showed a similar emission spectrum. Therefore, the observed fluorescence might be caused by the caged IPTG ester intermediates, the nitropiperonal uncaging product or a combination of both. Fluorescence intensities are not compared directly since the emission spectra were obtained with different devices and the recovery rate of the HPLC method for caged IPTG ester intermediate isolation was not determined. However, the course of the fluorescence intensity during the cultivation can still be valuable to characterize the uncaging reaction.

**Fig. 4:**
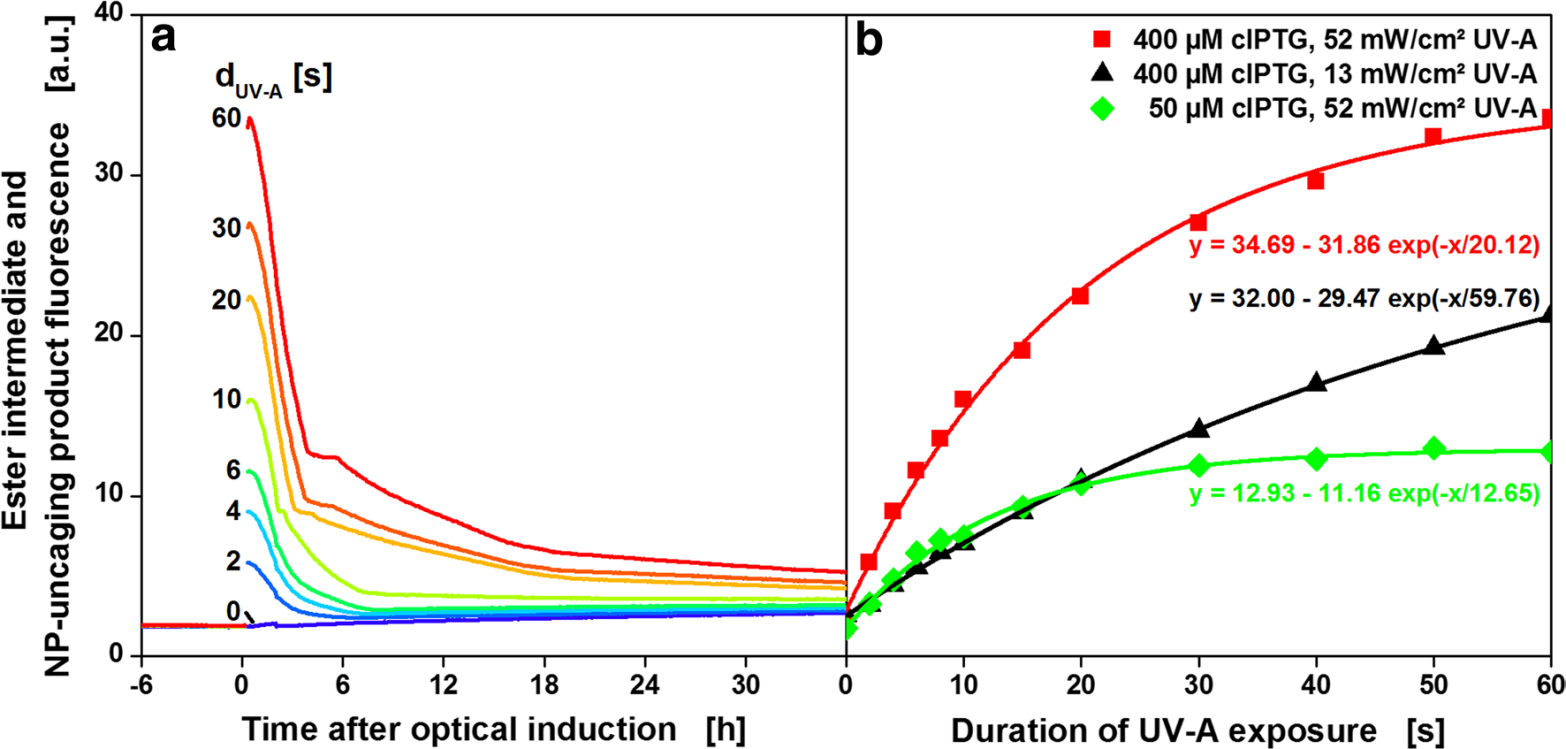
Online measurement of cIPTG ester intermediates and NP-uncaging product. Fluorescence intensity (λ_Ex_ = 326 nm, λ_Em_ = 407 nm, *black cross* in Fig. 2) of 12 *E. coli* cultures before and after UV-A irradiation for 0–60 s (**a**) and fluorescence intensity measured directly after irradiation as a function of duration of UV-A exposure (**b**). At the beginning of the cultivation, 400 µM cIPTG were added to the medium. After 10 h, optical induction was performed with the LED array (λ_max_ = 368 nm, I = 52mW/cm^2^). The amount of ester intermediates increases with increasing duration of UV-A exposure and can be fitted with first-order kinetics (*solid lines* and equations in B, R^2^ > 0.995). Reduced irradiance leads to lower rate constants (*black triangles*, I = 13 mW/cm^2^) and reduced cIPTG concentration to lower amplitude (*green diamonds*, 50 µM cIPTG). Cultivation conditions: 800 µL Wilms-MOPS mineral medium (20 g/L glucose, 0.2 M MOPS) per well in a 48-FlowerPlate, 30 °C, shaking frequency: 1000 rpm, shaking diameter: 3 mm. Not all data shown in **a** (complete set provided in Additional file 8)

For 12 cultures the duration of UV-A irradiation for optical induction was varied from 0–60 s (Fig. 4a). As a consequence of UV-A irradiation, the fluorescence signal rises instantly. Then, the fluorescence intensities quickly decrease over the course of the following 2–3 h (Fig. 4a). HPLC measurements confirmed that cIPTG ester intermediates were formed after UV-A irradiation. They were stable for at least 24 h when no cells were present, but fully degraded when lipase PL from *Alcaligenes sp.* was added (Additional file 3). This fits in well with previous reports where cIPTG ester intermediates are obtained within seconds to minutes of UV-A irradiation and are subsequently hydrolysed to the nitropiperonal uncaging product and IPTG within minutes to hours (t_1/2_ = 63 min) [15]. Possibly, the nitropiperonal uncaging product shows weaker fluorescence than the cIPTG ester intermediates and the fast decline of the fluorescence intensity in the first 2–3 h after irradiation indicates the conversion of cIPTG ester intermediates to the nitropiperonal uncaging product. The following slower signal decay over the remaining cultivation time could be caused by cell growth masking the fluorescence signal or subsequent reactions, e.g. dimerization, of the nitropiperonal uncaging product [21].

### Characterization of optical induction with cIPTG

Online fluorescence spectroscopy is already a method frequently applied to investigate other uncaging processes [22]. In Fig. 4b the ester intermediate and NP-uncaging product fluorescence measured directly after irradiation is given as a function of duration of exposure (d_UV-A_ = 0–60 s). With longer duration of exposure more cIPTG is uncaged and higher ester intermediate concentrations are detected. Since the substrate of the uncaging reaction (cIPTG) is depleted during the reaction the reaction rate decreases accordingly. The data are in very good accordance with first-order kinetics (equations and solid lines in Fig. 4b, R^2^ > 0.995). HPLC measurements confirmed the photo-uncaging of cIPTG as a function of UV-A exposure duration (Additional file 4). As to be expected for a photochemical reaction, the reaction rate constant is dependent on irradiance. This is shown by a reduction of irradiance by 75 % from 52 to 13 mW/cm^2^, resulting in a 66 % slower reaction (black triangles, Fig. 4b). The ester intermediate concentration is also influenced by the amount of cIPTG added at the beginning of the cultivation. A reduction of the initial cIPTG concentration by 87.5 % from 400 µM to 50 µM cIPTG reduces the ester intermediate signal by 63 % (green diamonds, Fig. 4b). These experiments show that three parameters can be used to control the amount of IPTG that is uncaged by optical induction: duration of UV-A exposure, irradiance and initial concentration of cIPTG. In the following experiments only duration of UV-A exposure (d_UV-A_ = 0–60 s) is used to control the amount of released IPTG while irradiance and initial cIPTG concentration are kept constant (52 mW/cm^2^, 400 µM cIPTG).

**Fig. 5:**
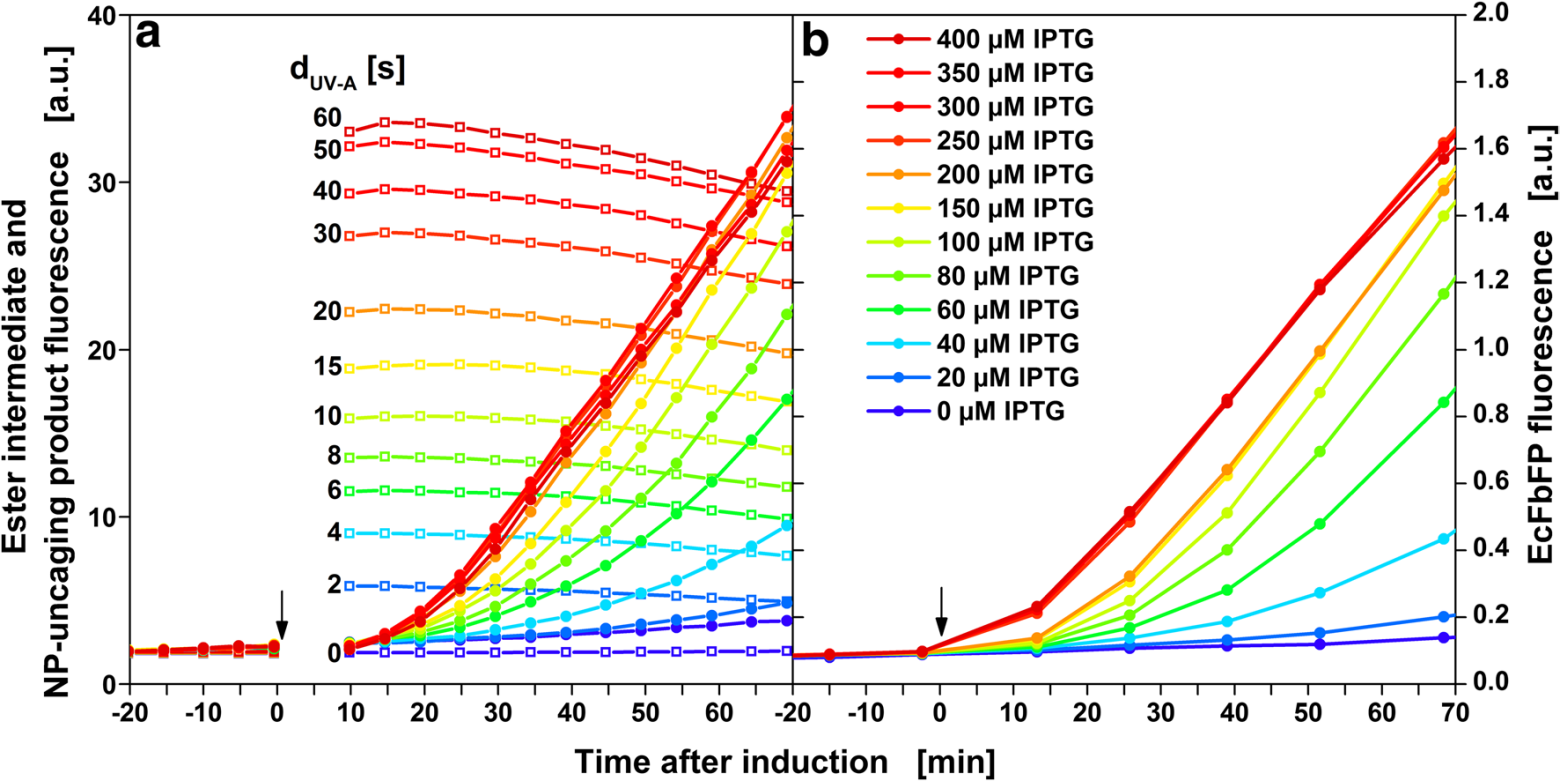
Onset of heterologous protein production after induction with cIPTG or IPTG. Fluorescence intensity of ester intermediate and NP-uncaging product (*open squares*, λ_Ex_ = 326 nm, λ_Em_ = 407 nm) and FbFP fluorescence intensity (*circles*, λ_Ex_ = 450 nm, λ_Em_ = 495 nm) immediately before and after induction with either cIPTG (**a**) or IPTG (**b**). Induction is performed with 0–60 s of UV-A irradiation or 0–400 µM IPTG after 10 h of cultivation. In **a** more data points per time are shown because fewer wells were monitored in parallel

For a comparison of optical induction with cIPTG to conventional induction with manually added IPTG solution, it is interesting to know how much of a delay is caused by the two-step uncaging mechanism. A significant delay would complicate a direct comparison of the two induction methods and would make a faster one-step uncaging mechanism more desirable. Figure 5a shows the development of fluorescence signal mediated by the formation of ester intermediates and FbFP immediately after optical induction. FbFP fluorescence before induction (black arrow) is very low which indicates that the expression system is tightly regulated so that, if at all, only minor amounts of target protein are produced. This furthermore substantiates that cIPTG added at the start of the cultivation does indeed not induce the cells prior to UV-A irradiation. After UV-A irradiation, the ester intermediate and NP-uncaging product signal increases instantly. Depending on the duration of UV-A exposure, an increase in FbFP fluorescence can be detected 15–25 min after optical induction (Fig. 5a). About the same delay is observed for conventional induction with IPTG solution (Fig. 5b). The observed response time after optical induction is much faster than reported in literature [23]. This means that under high irradiance conditions (52 mW/cm^2^) and with high initial cIPTG concentrations (400 µM cIPTG), used in this investigation, the uncaging mechanism of cIPTG is not the rate limiting step preceding transcription. Onset of protein formation seems to be equivalent for both methods of induction.

The FbFP signal also shows that the rate of product formation can be increased gradually with either increasing duration of UV-A exposure or increasing IPTG concentration. Both induction methods allow fine-tuning the initial product formation rate in the same range and to the same maximum. In this experiment, the maximum initial product formation rate is reached with either about 30 s UV-A or 250 µM IPTG.

A distinct advantage of the constructed UV-A LED array is that optical induction can obviously be achieved within seconds where previously several minutes of irradiation were required for uncaging [15, 19]. The very short induction times are most probably a result of the high irradiance of 52 mW/cm^2^. Since the strong UV-A irradiation applied in our design could be potentially phototoxic we tested the influence on non-induced cultures. For exposure durations of up to 60 s, no influence on biomass growth was observed during the exponential phase and only minute deviations were detected in the stationary phase (Additional file 5). Therefore, phototoxicity due to UV-A irradiation is of no concern for the short UV-A exposure times (d_UV-A_ = 0–40 s) applied in the subsequent experiments.

### Induction profiling

**Fig. 6:**
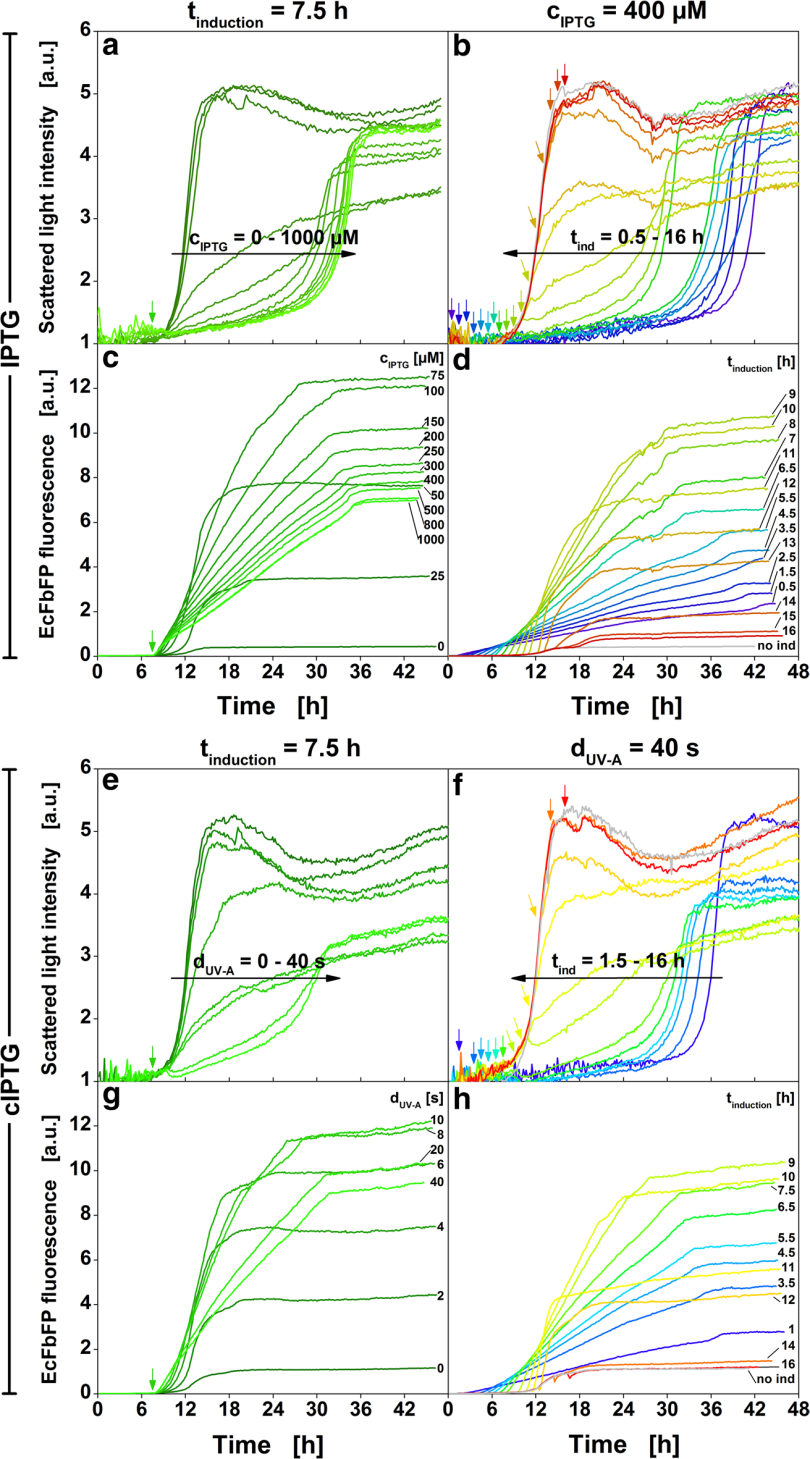
Online measurement data for conventional induction profiling with manual addition of IPTG solution and optical induction profiling with cIPTG. Scattered light and FbFP fluorescence of cultures induced with IPTG (**a**–**d**) and cultures induced with cIPTG (**e**–**h**). Time of induction and inducer strength (IPTG concentration or duration of UV-A exposure) were varied in full factorial design. Colors from *blue* to *red* mark later induction times (0.5–16 h), dull to bright colors mark increasing inducer strength (0–1000 µM IPTG or 0–40 s duration of UV-A exposure). In total, 304 cultures were induced conventionally and 96 cultures were induced optically. The first column (**a**,** c**,** e**, **g**) shows a subset of cultivations with a fixed induction time of 7.5 h and the second column (**b**, **d**, **f**, **h**) shows a subset of cultivations with a fixed inducer strength of 400 µM IPTG or 400 µM cIPTG and 40 s UV-A exposure. The online signals for all 400 cultivations are provided in Additional file 9. *Small colored down*-*pointing arrows* illustrate the time of induction (not all shown). *Long horizontal arrows in black* illustrate general trends, e.g. impact of increasing inducer concentration on growth (**a**). Cultivation conditions: 800 µL Wilms-MOPS mineral medium per well in a 48-FlowerPlate, 400 µM cIPTG added to cultures induced with the LED array (λ_max_ = 368 nm, I = 52 mW/cm^2^), 30 °C, shaking frequency: 1000 rpm, shaking diameter: 3 mm

So far, optical induction with cIPTG was shown to be a robust method that allows gradual inducer release, very fast onset of protein synthesis and fine-tuning of protein formation. Since protein formation was essentially equal to conventional induction the optical induction method was applied for detailed induction profiling with variation of time of induction and duration of UV-A exposure to maximize product formation. For comparison, conventional induction profiling with manual addition of IPTG solution was conducted as well. Online signals for biomass (scattered light) and FbFP fluorescence are given in Fig. 6. Induced cultures differ from non-induced cultures in growth and product formation after the respective time of induction (indicated by arrows). A number of trends can be deduced from the experiments as discussed below.

Firstly, increasing inducer concentrations reduce growth rates and delay the time to reach final biomass concentration (Fig. 6a, e). For example, a non-induced culture reaches the maximum biomass signal (scattered light intensity) after 18 h while a culture induced with 400 µM IPTG reaches the maximum 19 h later (Fig. 6a, time of induction: 7.5 h). A growth delay of about 17 h is observed when 400 µM of cIPTG are irradiated with UV-A light for 40 s at the same time point (Fig. 6e).

Secondly, a delay in growth is also observed when inductions are performed earlier rather than later (Fig. 6b, f). For example, the highest biomass signal is reached after about 40 h if the induction is performed 1.5 h into the cultivation with either 400 µM IPTG (Fig. 6b) or 400 µM cIPTG and 40 s of UV-A light (Fig. 6f). For later inductions this effect is gradually less pronounced because the cultivation has already progressed further. Decreased growth rates are caused by the drain of metabolites into heterologous protein formation (“metabolic burden”) and models to correlate growth rate and heterologous protein formation have been developed [4, 24, 25].

Thirdly, initial product formation is faster with higher inducer concentrations (Fig. 6c, g; a more detailed view is provided in Additional file 6). However, over the course of the following production phase the rate decreases slightly with more pronounced decreases for higher IPTG concentration (150–1000 µM). This decrease might be attributed to overloading protein synthesis capabilities with gratuitous mRNA coding for the target protein and thereby, in the long run, decreasing synthesis capability. In contrast, cultures induced with less IPTG (25–100 µM) and lower initial product formation rates are less strained and (as visible from the biomass signal) still exhibit growth. Therefore, their production rates can still increase and eventually surpass those of more strongly induced cultures. High product concentrations at the end of the cultivation are not reached with high IPTG concentrations, but rather with moderate values of e.g. 75 to 100 µM (Fig. 6c) or 8–10 s UV-A light (Fig. 6g) (when the induction is preformed 7.5 h after the start of the cultivation).

Fourthly, product formation is also influenced by the time of induction. If, for example, the induction is performed with rather high IPTG concentrations (400 µM, Fig. 6d) or long exposures (40 s, Fig. 6h), product concentration is highest, if the induction is performed 9 h after inoculation. For earlier induction times not enough cells are present to produce high amounts of protein, for later induction times not enough substrate is left.

**Fig. 7:**
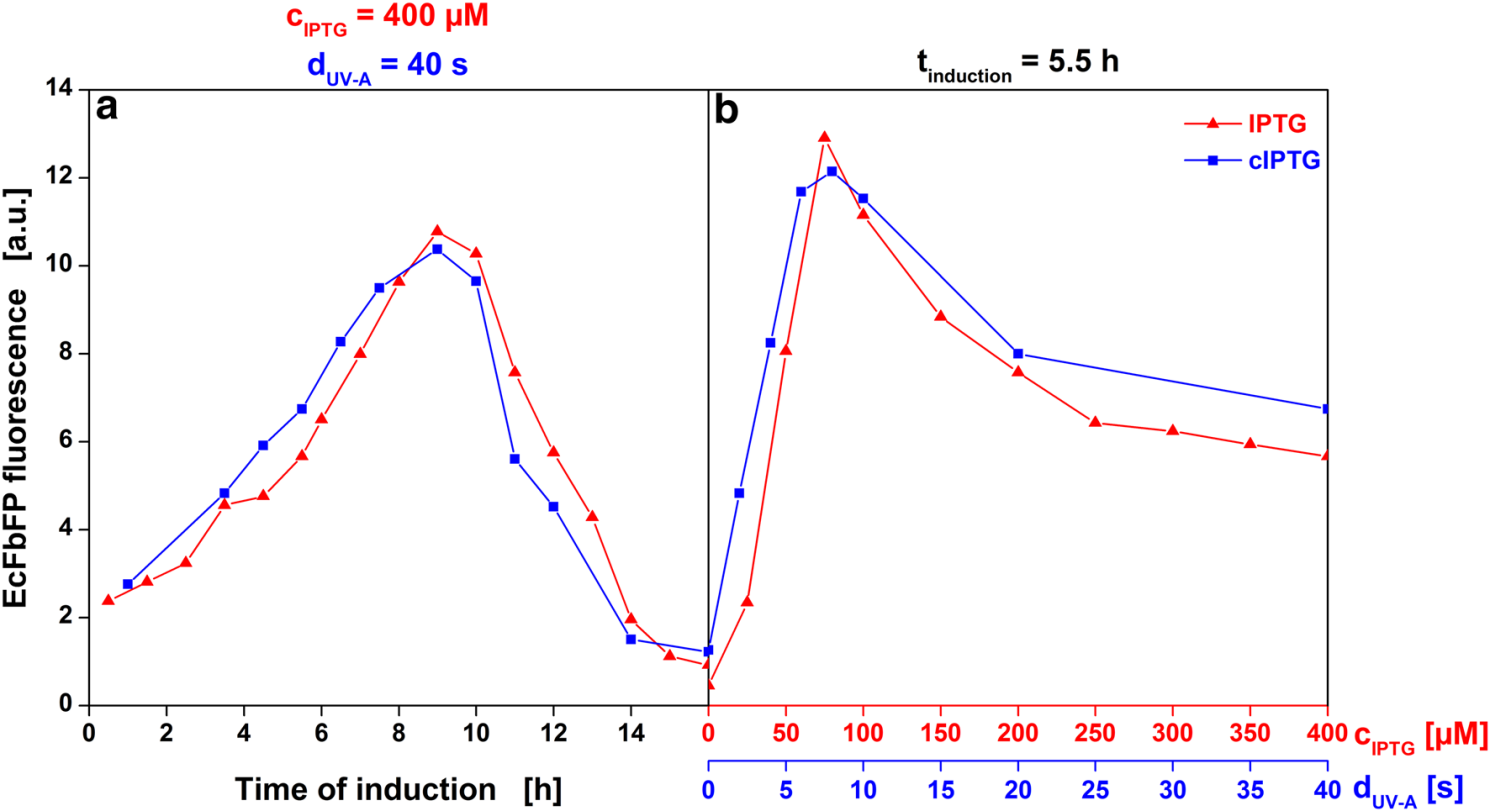
Comparison of end-point FbFP fluorescence after induction with IPTG or cIPTG. FbFP fluorescence after 42 h of individual cultures induced at varied time points (0.5–16 h) with either 400 µM IPTG (*red curve*) or cIPTG after 40 s UV-A exposure (*blue curve*) (**a**) and of cultures induced after 5.5 h with either 0–400 µM IPTG or 0–40 s UV-A exposure (**b**)

In summary, the microtiter plate-based approach allowed to collect not just end-point but quasi-continuous data of high resolution and over a broad range of induction conditions. Thereby, it could be shown that the observed effects of conventional and optical induction on growth and product formation are equivalent under the applied conditions. This is demonstrated even more clearly in Fig. 7 which exclusively shows end-point fluorescence measured after 42 h of cultures induced either conventionally (red) or optically (blue). For a constant inducer concentration (400 µM IPTG or 40 s UV-A exposure) the impact of induction time is shown in Fig. 7a. Vice versa, for a constant induction time (5.5 h) the impact of inducer concentration is shown in Fig. 7b. The trends and maxima of target protein concentration are once again very similar for both induction methods. Further studies are needed to explain the slight shift of about 30 min to earlier induction times for optical induction shown in Fig. 7a. It may result from either the two-step uncaging mechanism or from experimental deviations between consecutive cultivations. As already demonstrated in Fig. 5, the hydrolysis of the caged IPTG ester intermediates by intracellular esterases does not represent the rate limiting step under the conditions applied in this work.

Figure 7 demonstrates that induction conditions optimized with either method can be applied interchangeably. The transfer of an optimized operation point including time of induction and inducer concentration or duration of UV-A exposure, respectively, is straight-forward: Time of induction is about equivalent and duration of UV-A exposure is, in first approximation, linearly correlated to IPTG concentration with 10 s of UV-A irradiation being equivalent to 100 µM IPTG under the applied conditions (52 mW/cm^2^, 400 µM cIPTG). HPLC–UV measurements confirmed that 116.2 µM (±29.4 µM) cIPTG were uncaged under these conditions (Additional file 4). However, a direct quantification of the subsequently released IPTG was not possible due the detection being more difficult. Based on the reaction scheme by Young and Deiters the amount of cIPTG uncaged to ester intermediates and the amount of subsequently released IPTG are expected to be equivalent (Fig. 3a). Further experiments are required to independently verify the amount of released IPTG. Fernández et al. developed an HPLC–MS method for the direct quantification of intracellular IPTG in the required µM range [26]. Further experiments would also be required to construct a more detailed model that describes the amount of released IPTG as a function of all optical parameters (UV-A exposure duration, irradiance, cIPTG concentration) and first-order kinetics should be considered for the impact of UV-A exposure duration as presented in the fits in Fig. 4b and Additional file 4.

**Fig. 8:**
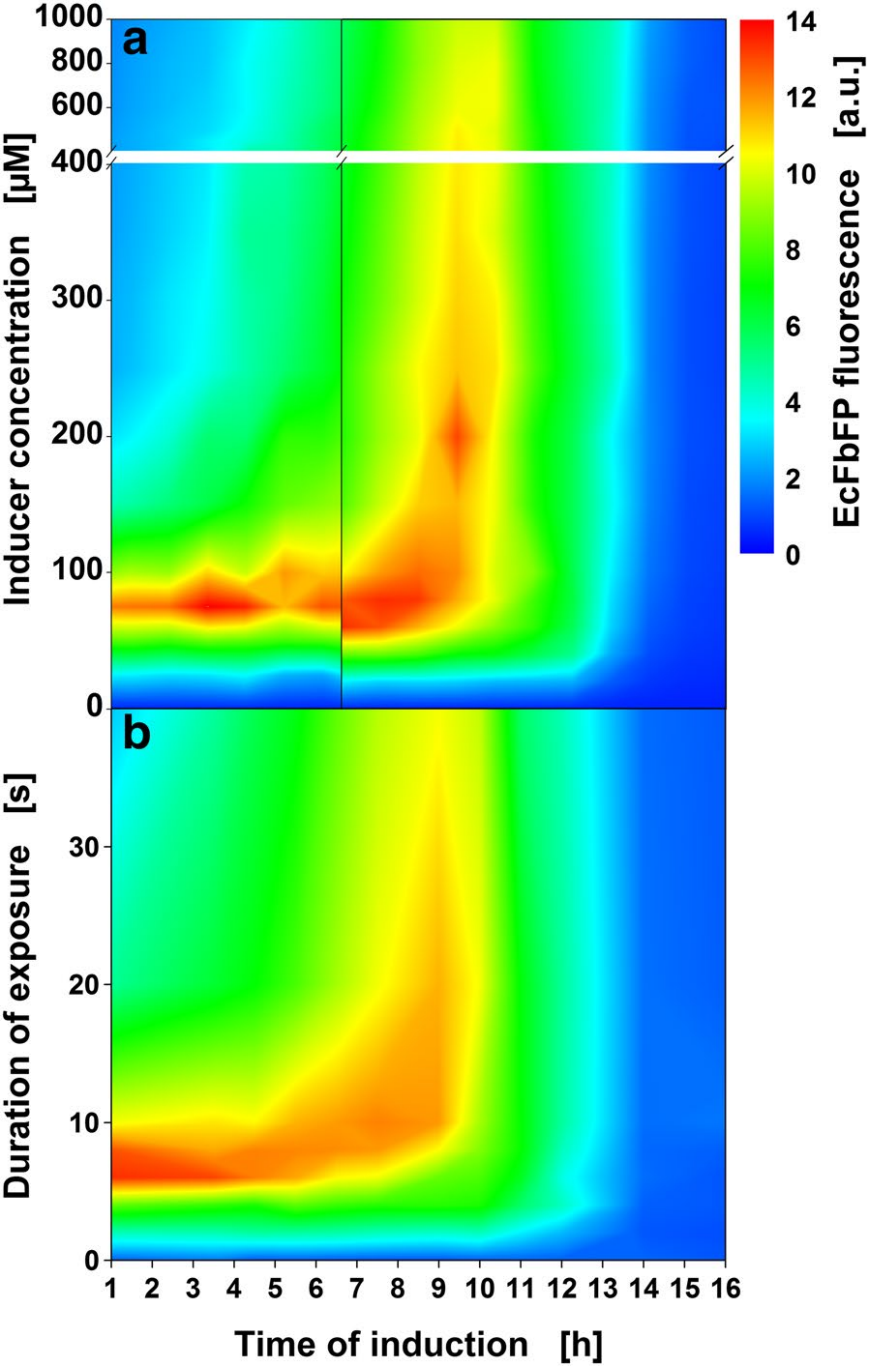
Induction profiles for conventional induction with manual addition of IPTG solution and optical induction with cIPTG. End-point FbFP fluorescence of cultures conventionally induced with IPTG (**a**) or optically induced with cIPTG upon UV-A exposure (**b**). Colors from *blue* to *red* indicate induction conditions that lead to increasing FbFP concentrations. For optical induction 400 µM cIPTG was added at the start of the cultivation. Note the axis break at 400 µM IPTG in so that both induction profiles are scaled equally in the range of 0–400 µM IPTG and 0–40 s UV-A exposure to facilitate comparison (**a**). Results for higher IPTG concentrations (400–1000 µM) are still shown but do not yield higher FbFP concentrations. Also note the vertical black line at an induction time of 6.5 h that highlights a change in microtiter plate lot. IPTG induction experiments with induction times of 1–6.5 h were performed in 48-FlowerPlates of lot 14xx, all other experiments were performed with plates of lot 15xx. The normalization procedure is described in Additional file 7. Cultivation conditions for *E. coli* Tuner(DE3)/pRhotHi-2-LacI-EcFbFP: 800 µL Wilms-MOPS mineral medium per well in a 48-FlowerPlate, 30 °C, shaking frequency: 1000 rpm, shaking diameter: 3 mm

The operating point for highest target protein concentration can be chosen from Fig. 8 which shows FbFP fluorescence at the end of all induction profiling cultivations. This plot reveals the existence of two general induction regimes that can be applied to achieve high target protein concentrations. If the time of induction is set to 9 h, a broad range of IPTG concentrations (100–400 µM) or UV-A exposure durations (10–40 s) leads to good product formation represented by yellow to red colors in a vertical zone. A second zone with slightly higher protein concentrations is oriented horizontally and represents the optimum IPTG concentration (60–80 µM) or UV-A exposure duration (6–8 s) for early induction. Within this region, time of induction only has a minor influence (1–6 h).

The presented induction profiles are in accordance with data on a closely related strain (*E. coli* BL21(DE3)/pRhotHi-2-EcFbFP) cultivated in the same medium, previously published by Huber et al. [9]. Since in this work four microtiter plates were monitored in parallel instead of just one, induction profiles with more data points and higher resolution could be obtained. The importance of detailed induction profiling with high resolution can be exemplified by deviations from the optimum IPTG concentration in the horizontal regime. Inductions with 50 or 150 µM IPTG at 2.5 h yield only 60 % of the protein that is produced after induction with 75 µM IPTG. The still prevalent induction with 1000 µM IPTG in the early exponential growth phase [2] (in our case after approx. 9–10 h) is also far from ideal to achieve high target protein concentrations with the strain used in this work. The highest protein concentration after an induction with 1000 µM IPTG is achieved when the culture is induced after 10 h. It yields only 70 % of the target protein that is achieved when the culture is induced with 75 µM IPTG after 2.5 h. Thus, overinduction with too much IPTG not only wastes a cost-intensive chemical but also reduces product concentration. An efficient process with high product concentration can only be reached when induction parameters are optimized for each individual strain and target protein. Light-induced gene expression with photocaged IPTG applied in a high-throughput system allows performing this optimization much more easily than before.

## Conclusions

An LED array allowing for individual illumination of each well of a 48-well microtiter plate was constructed and successfully applied for light-induced gene expression based on photocaged IPTG. The fluorescence of the reporter protein FbFP was measured online in up to four microtiter plates in parallel. Irradiating photocaged IPTG resulted in an additional distinct fluorescence signal which could be assigned to photocaged IPTG ester intermediates and the nitropiperonal uncaging product. This signal was subsequently applied to characterize the uncaging reaction. In the future, it might also be utilized to control more advanced induction strategies. As an example, the IPTG concentration profile over time which usually follows a step function (addition or release at one time point) could be replaced by fine-tuned profiles with continuous inducer release depending on current biomass concentration or growth rate [27–29]. The combination of an LED array and a BioLector screening system could also be used to study gene expression and protein production controlled by chemically distinct photocaged compounds or by photoreceptors [30–32].

In this study, we compared optical induction with photocaged IPTG to conventional induction with manual addition of IPTG solutions and showed that both methods yield essentially equivalent results under the applied conditions. This means that induction parameters which can be optimized easily with optical induction can later be transferred to conventional induction protocols for e.g. stirred tank bioreactors. Optical induction can be parallelized easily as well and it has the distinct advantage of being less invasive as the shaking motion of the microtiter plate is not stopped. This is of great importance since even short oxygen limitation influences expression of genes involved in central metabolism and can lead to a complete loss of productivity in organisms sensitive to sudden oxygen limitation [33, 34]. Also, optical measurements which are affected by a change in the shaking motion can be continued throughout the induction. Furthermore, the sterile barrier is not compromised by optical induction which might prove very convenient for strictly anaerobic cultivations or cell culture applications [35]. In conclusion, light-induced induction shows several advantages and can readily replace conventional induction for screening purposes in microtiter plates.

## Methods

### Microorganism and target protein

The strain *Escherichia coli* Tuner(DE3)/pRhotHi-2-LacI-EcFbFP was used for all experiments. Except for the target protein it is identical to a strain previously used to study light-induced gene expression by Binder et al. [19]. It offers several advantageous characteristics for this study: Because of the permease deficiency (*lacY*^−^) of *E. coli* Tuner(DE3) IPTG can only enter the cell via concentration dependent diffusion. Therefore, an unimodal induction response homogenously distributed over the entire population can be expected [19, 36]. The low-copy number plasmid pRhotHi-2 carrying a T7 promoter offers potential for high-level expression [37]. Additional expression of the repressor LacI reduces the basal expression which allows easier quantification at low induction levels [19]. The flavin-based fluorescent protein FbFP was chosen as the target protein because its fluorescence develops immediately after translation and no maturation step depending on intracellular oxygen concentration is required as for proteins of the GFP-family [38]. For better expression, a sequence optimized for *E. coli* codon bias was used [20].

The construction of expression vectors and recombinant DNA techniques were carried out in *E. coli* DH5α [39] as described by Sambrook et al. [40]. The EcFbFP reporter gene was isolated from pRhotHi-2-EcFbFP [37, 41] and inserted into target vector pRhotHi-2-LacI via *Nde*I/*Xho*I restriction, yielding the final construct pRhotHi-2-LacI-EcFbFP.

### Precultivations

Prior to induction experiments two sequential precultivation steps were performed. The first precultivation step was done with 10 mL complex TB medium (5 g/L glycerol, 24 g/L yeast extract, 12 g/L tryptone, 12.54 g/L K_2_HPO_4_, 2.3 g/L KH_2_PO_4_; all ingredients from Roth, Germany) in 250 mL shake flasks. Shake flasks were inoculated to an initial optical density of 0.1 (OD_600_) from cryogenically preserved cultures and cultivated on an orbital shaker (LS-X, Kuhner, Switzerland) at 350 rpm with a shaking diameter of 50 mm for 5 h at 37 °C. For the second precultivation step 10 mL of a modified Wilms-MOPS minimal medium [42] (20 g/L glucose, 6.98 g/L (NH_4_)_2_SO_4_, 3 g/L K_2_HPO_4_, 2 g/L Na_2_SO_4_, 41.85 g/L (N-morpholino)-propanesulfonic acid (MOPS), 0.5 g/L MgSO4·7H_2_O, 0.01 g/L thiamine hydrochloride, 1 mL/L trace element solution [0.54 g/L ZnSO_4_·7H_2_O, 0.48 g/L CuSO_4_·5H_2_O, 0.3 g/L MnSO_4_·H_2_O, 0.54 g/L CoCl_2_·6H_2_O, 41.76 g/L FeCl_3_·6H_2_O, 1.98 g/L CaCl_2_·2H_2_O, 33.4 g/L Na_2_EDTA (Titriplex III)]) were used. The pH-value was adjusted to 7.5 with NaOH. Shake flasks were again inoculated to an initial optical density of 0.1 (OD_600_) and placed on an orbital shaker at 350 rpm with a shaking diameter of 50 mm. After cultivation for 9 h at 30 °C the second precultures reached the early exponential growth phase and the main culture in microtiter plates was inoculated. In all cultivation steps 50 µg/mL kanamycin sulfate was added.

### Microtiter plate cultivations

Microtiter plate cultivations for induction experiments were conducted in 48-well FlowerPlates (MTP-48-B, lot 1404 & 1509, m2p-labs, Germany). As in the second precultivation step Wilms-MOPS mineral medium was used. Wilms-MOPS medium is suited to achieve high cell densities and high heterologous protein concentrations [42]. As demonstrated elsewhere, optical induction with cIPTG is not restricted to mineral media and can also be performed in complex media like LB [15, 19]. LB medium was not used here because it is restricted to low cell densities since its nitrogen-containing complex compounds are used as energy source which results in the formation of ammonium and alkalization [43]. Other complex media like TB were not used because lot-to-lot variations in raw materials have been reported to effect reproducibility in induced cultures [44]. Each well was filled with 800 µL Wilms-MOPS medium from a master mix inoculated to an initial optical density of 0.1 (OD_600_). Plates were sealed with an autoclaved self-adhesive transparent polyolefin sealing foil (900371, HJ-Bioanalytik, Germany) as sterile barrier. The foil reduces evaporation while still allowing sufficient gas transfer. Up to four microtiter plates in parallel were placed on an orbital shaker (ES-X, Kuhner, Switzerland) with a shaking frequency of 1000 rpm and a shaking diameter of 3 mm at 30 °C.

### Optical induction with LED array

Nitropiperonal-photocaged IPTG (cIPTG) was synthesized from IPTG and 6-nitropiperonal as previously described [19]. At the beginning of microtiter plate cultivations for optical induction 400 µM cIPTG was added from a 40 mM stock solution in DMSO stored in the dark at −20 °C. An in-house constructed array of 48 high-power UV-A LEDs (LZ1-3x, LED Engin, USA) attached to a heat sink was mounted on top of a microtiter plate resulting in one LED per well to be positioned at a distance of 14 mm in coaxial position (Fig. 1). A mask with cylindrical holes with 11 mm diameter ensures that only the corresponding well is illuminated and no stray light can enter adjacent wells. No induction was observed in wells that were adjacent to illuminated wells but not illuminated themselves. Between heat sink and mask a 3 mm gap is left to allow sufficient gas transfer to and from the wells. When an LED is switched on, UV-A irradiation passes the transparent sterile barrier and reaches the culture where IPTG is uncaged. LEDs are wired in a matrix layout and switched using 14 metal oxide semiconductor field-effect transistors on a driver stage fixed onto the shaking tray next to the microtiter plate. The driver stage is connected to a microcontroller (Arduino Uno R3, Arduino LLC, USA) and controlled via a PC running LabVIEW (LabVIEW v14, National Instruments, USA). LEDs are operated at 700 mA resulting in a high irradiance of at the well bottom (52.7 mW/cm^2^, λ_max_ = 368 nm) with low well-to-well deviation (SD 3.2 mW/cm^2^). Irradiance at the position of the well bottom was determined on bottomless microtiter plates with a thermal power sensor (S302C, Thorlabs, Germany). For optical induction experiments cultures were irradiated for 2–60 s. During irradiation a temperature increase of 0.7 K/min occurred which was measured using a rhodamin-based fluorescence method [45, 46] (data not shown). Since uncaging was achieved within 1 min, the temporary temperature increase (<0.7 K) was considered to be within acceptable limits. For one of the experiments performed to characterize photo-uncaging a lower effective irradiance (13 mW/cm^2^) was used. This was achieved by placing a strip of diffuser foil (White Diffusion LEE216, LEE Filters, USA) directly below the LEDs.

### Fluorescence spectroscopy

Fluorescence measurements were performed through the transparent bottom of the microtiter plates during cultivation, according to the established BioLector setup [11, 17]. A quartz/quartz multi-mode fiber (LUV 105 µM, LEONI, Germany) is moved sequentially below the wells of four microtiter plates by a Cartesian motion system (CMS, Bosch Rexroth, Germany) to allow quasi-continuous fluorescence measurements on all wells without stopping the shaking movement which might otherwise result in cell sedimentation or oxygen limitation. In contrast to commercially available designs, a spectrofluorometer with excitation/emission monochromators (Fluoromax-4, HORIBA Jobin–Yvon GmbH, Germany) was applied. It enables variation and optimization of excitation/emission wavelength in a range of 200–950 nm. During cultivation cIPTG ester intermediates were excited at a wavelength of λ_Ex_ = 326 nm and therefore below the maximum excitation wavelength of λ_Ex.max_ = 335 nm to reduce NADH fluorescence crosstalk. cIPTG ester intermediate fluorescence was measured at λ_Em_ = 407 nm. FbFP fluorescence (λ_Ex_ = 450 nm, λ_Em_ = 495 nm) and biomass formation (scattered light at 650 nm) were monitored as well. When measuring the cIPTG ester intermediate fluorescence the excitation and emission slits were set to a bandwidth of 8 nm. The raw intensities measured for FbFP fluorescence and biomass (scattered light) were higher and therefore the slit bandwidth could be reduced to 4 nm. Integration time was set to 600 ms for fluorescence signals and 900 ms for scattered light (biomass). 2D fluorescence spectra of cultures before and after uncaging with UV-A irradiation were obtained by scanning the excitation wavelength from 300 to 600 nm and the emission wavelength from 320 to 600 nm (stepsize: 5 nm) according to a previously published setup [18]. As reference, cIPTG ester intermediates and the nitropiperonal-uncaging product were isolated by HPLC (Additional file 1), identified by NMR (Additional file 2) and fluorescence spectra from 360 to 500 nm were obtained with a plate reader (λ_Ex_ = 330 nm, Infinite M1000 Pro, Tecan, Switzerland).

### Induction profiling

Conventional induction with IPTG and optical induction with uncaging of cIPTG were compared in induction experiments. A culture well was either conventionally induced by manually adding IPTG solution with a pipette or optically induced by illumination with the LED array. The two parameters varied were time of induction (in relation to the start of the cultivation, 0.5–16 h) and either IPTG concentration (0–1000 µM) in case of conventional induction or duration of UV-A exposure (0–40 s) in case of optical induction. Conventional induction was achieved by pausing the measurement, stopping the shaker, removing the sealing foil and manually adding 20 µL IPTG solution from a set of sterile stock solutions with a multichannel pipette (Eppendorf, Germany). Then, the sealing foil was replaced and the shaker was started again. For optical induction a cIPTG concentration of 400 µM and a high irradiance of 52.7 mW/cm^2^ were used. No manual interference was required at all since the LED array was automatically controlled by a LabVIEW script during shaking.

During the induction profiling experiments the microtiter plate supplier (m2p-labs, Germany) changed the manufacturing of the plates’ transparent bottom. According to the supplier, for FlowerPlates with lot numbers 14xx and earlier a 150 µM thin polystyrene foil (158 K, BASF, Germany) was used. In contrast, a 700 µM thick polystyrene foil (Styron 678E, Dow Chemical, USA) was used for plates with lot number 15xx and later. The thicker foil reduced fluorescence and scattered light intensities. Also, the plates with higher lot numbers feature two small opaque spots (intended to take up pH- and oxygen sensor dyes) which increase the scattered light baseline. To allow comparison of cultures on different microtiter plates, data was normalized to the biomass signal (scattered light) of non-induced reference cultures (Additional file 7).

## Abbreviations

IPTG: isopropyl β-d-1-thiogalactopyranoside; cIPTG: 6-nitropiperonal-photocaged isopropyl β-d-1-thiogalactopyranoside; FbFP: flavin-mononucleotide-based fluorescent reporter protein; T7-RNA-polymerase: ribonucleic acid polymerase from bacteriophage T7; *E. coli*: *Escherichia coli*; UV-A: ultraviolet A radiation with a wavelength of 315–400 nm; LED: light-emitting diode; 2D: two-dimensional; EcFbFP: *E. coli* codon bias-optimized FbFP; HPLC: high-performance liquid chromatography; NMR: nuclear magnetic resonance spectroscopy; GFP: green fluorescent protein; DNA: deoxyribonucleic acid; TB: terrific broth; MOPS: (*N*-morpholino)-propanesulfonic acid; EDTA: ethylenediaminetetraacetic acid; DMSO: dimethyl sulfoxide; SD: standard deviation; NADH: reduced form of nicotinamide adenine dinucleotide; NP: 6-nitropiperonal; cIPTGe: ester intermediate in the cIPTG photouncaging reaction.

### List of symbols

λ_max_: wavelength with maximum intensity; OD_600_: optical density at a wavelength of 600 nm; λ_ex,max_: excitation wavelength resulting in maximum fluorescence emission; λ_em,max_: wavelength with maximum fluorescence emission; λ_ex_, λ_em_: fluorescence excitation and emission wavelength; t_1/2_: half-life; d_UV-A_: duration of UV-A exposure; I: irradiance; t_induction_: time of induction; c_IPTG_: IPTG concentration.

## Additional files

10.1186/s12934-016-0461-3 cIPTG was dissolved in isopropanol/n-heptan 50/50. (8.3 mg in 2.5 mL) and irradiated for 10 min (375 nm; 6.2 mW/cm²). cIPTG and its ester intermediates (cIPTGe1 and cIPTGe2) were then separated via HPLC (column: Chiralpak IC, 250·10 mm, Daicel, Japan; solvent: n-heptan:2-propanol (30:70); flow rate: 0.5 mL/min; detection: UV 258 nm)
10.1186/s12934-016-0461-3. NMR-measurement of ester intermediates. cIPTGe1 (A) and cIPTGe2 (B) were identified via NMR. **cIPTGe1 (A):** 1H-NMR (600 MHz, CDCl3), δ [ppm]: 7.35 (s, 1 H, 4´-CH), 6.01 (s, 1 H, 7´-CH), 6.14 (s, 2 H, 2´-CH2), 4.75 (dd, 2J6a, 6b = 11.5 Hz, 2J6a, 5 = 6.0 Hz, 1 H, 6-CH2), 4.66 (dd, 2J6b, 6a = 11.4 Hz, 2J6b, 5 = 6.6 Hz, 1 H, 6-CH2), 4.39 (m, 1 H, 1-CH), 3.98 (m, 1 H, 4-CH), 3.87 (m, 1 H, 5-CH), 3.63 (m, 2 H, 2-CH, 3-CH), 3.17 (septet, 3JSCH, CH3a/b = 6.70 Hz, 1 H, -SCH), 2.73 (s, 1 H, OH), 2.59 (s, 1 H, OH), 2.45 (s, 1 H, OH), 1.31 (d, 3JCH3a, SCH = 1.70 Hz, 3H, -CH3a), 1.30 (d, 3JCH3b, SCH = 1.80 Hz, 3H,-CH3b). 13C-NMR (151 MHz, CDCl3), δ [ppm]: 166.94 (C-7), 160.67 (C-6´), 153.16 (C-7a´), 150.80 (C-3a´), 133.61 (C-5´), 108.72 (C-4´), 103.36 (C-2´), 89.36 (C-7´), 86.00 (C-1), 75.66 (C-5), 74.34 (C-3), 70.51 (C-2), 68.47 (C-4), 64.69 (C-6), 35.92 (SCH), 24.22 (C- CH3a), 23.99 (C- CH3b). **cIPTGe2 (B):** 1H-NMR (600 MHz, CDCl3), δ [ppm]: 7.42 (s, 1 H, 4´-CH), 6.19 (s, 2 H, 2´-CH2), 6.01 (s, 1 H, 7´-CH), 5.69 (dd, 3J4,3 = 3.6 Hz, 3J4,5 = 1.1 Hz 1 H, 4–CH), 4.47 (d, 3J1,2 = 9.7 Hz, 1 H, 1-CH), 3.86 (m, 1 H, 5-CH), 3,78 (m, 2 H, 6a-CH2, 3-CH), 3.70 (dd, 3J6b, 6a = 11.9 Hz, 3J6b, 5 = 7.1 Hz, 1H, 6-CH2), 3.30 (t, 3J2,1 = 9.4 Hz, 3J2,3 = 9.4 Hz, 1 H, 2-CH2)), 3.18 (m, 1 H, -SCH), 1.31 (d, 3JCH3a/b, SCH = 1.6 Hz, 3H,-CH3a), 1.30 (d, 3JCH3a/b, SCH = 1.5 Hz, 3H,-CH3b)
10.1186/s12934-016-0461-3 Relative amount of cIPTG ester intermediates over time. No ester intermediates are detected without UV-A irradiation (-UV-A). After UV-A irradiation (+UV-A) ester intermediates are detected. They were stable for at least 24 h (+24 h). Addition of lipase PL from *Alcaligenes sp.* fully degrades the ester intermediates (+lipase). HPLC (Jasco HPLC system, column: Hyperclone 5 μ ODS (C18) 120 (Phenomenex), solvent: MeOH:H2O 30:70, flow rate: 1 mL/min, 25 °C, 30 μL, detection: UV 258 nm at 11.46 min). 1000 μM cIPTG in H_2_O, irradiation with 6.4 mW/cm² at 375 nm for 10 min and storage at RT for 24 h, addition of 1 mg lipase PL (Alcaligenes sp. lipase 100000 U/g) to 910 μL at 38 °C for 24 h
10.1186/s12934-016-0461-3 Photo-uncaging of cIPTG as a function of UV-A exposure duration. In vitro decomposition of 400 μM cIPTG in H_2_O by UV-A irradiation (λ_max_ = 368 nm, I = 52 mW/cm², n = 4) monitored via HPLC–UV. HPLC (Jasco HPLC system, column: Hyperclone 5 μ ODS (C18) 120 (Phenomenex), solvent: MeOH:H2O 30:70, flow rate: 1 mL/min, 25 °C, 30 μL, detection: UV 258 nm at 19.04 min)
10.1186/s12934-016-0461-3 Effect of UV-A irradiation on cell growth. Scattered light intensity of non-induced cultures irradiated with UV-A LEDs for 0–120 s (λ_max_ = 368 nm, I = 52 mW/cm²). No cIPTG was added to the medium. The black arrow indicates the time of UV-A exposure in the exponential phase. For up to 60 s of UV-A exposure only minute deviations are detected in the scattered light signal. Exposure for 120 s leads to a slightly lower scattered light signal in the stationary phase. Since exposure durations of up to 40 s were sufficient for optical induction, negative effects of UV-A irradiation are of no concern for the bacteria used in this work. Cultivations were performed in triplicates; standard deviation is shown in the same color as the mean value but at 50 % transparency
10.1186/s12934-016-0461-3 Initial product formation after induction. Zoomed view of Fig. 6c and Fig. 6g. FbFP fluorescence of *E. coli* cultures induced after 7.5 h with 0–1000 μM IPTG (A) or 400 μM of cIPTG and 0–40 s of UV-A irradiation (B). The initial product formation gradually increases with increasing IPTG concentrations (0–400 μM) and is saturated for higher concentrations (400–1000 μM) (A). However, the highest product fluorescence at the end of the cultivation after 42 h is reached with 75–100 μM IPTG (A, right side). For optical induction, initial product formation rate is highest for 20–40 s of UV-A irradiation and the highest product concentrations after 42 h are reached with 8–10 s (B). Note the axis scaling and breaks for increased readability. Additionally, note that only 400 μM of cIPTG are available for uncaging in B. Cultivation conditions: 800 μL Wilms-MOPS mineral medium per well in a 48-FlowerPlate, 400 μM cIPTG added to cultures induced with the LED array (λ_max_ = 368 nm, I = 52 mW/cm²), 30 °C, shaking frequency: 1000 rpm, shaking diameter: 3 mm
10.1186/s12934-016-0461-3 Data normalization. Raw scattered light signals are influenced by the microtiter plate lot (A). For normalization the raw signals of cultivations in one lot can be multiplied with a correction factor to match the course of the other cultivation (B). The correction factor is determined by dividing the scattered light intensities at the end of the cultivation after 42 h. This correction factor can also be applied to correct EcFbFP fluorescence signals (C). The normalized signals of cultures induced with 0, 100 or 200 μM IPTG are in good agreement. This demonstrates that reproducible results can be obtained even when different microtiter plate lots are used. Cultivation conditions: 800 μL Wilms-MOPS mineral medium per well in a 48-FlowerPlate, 30 °C, shaking frequency: 1000 rpm, shaking diameter: 3 mm. Error bars in A and B indicate the standard deviation of six reference cultures. Induction in C after 6 h. Data for 0 μM IPTG and lot 14xx is not visible in C because it is almost identical to lot 15xx
10.1186/s12934-016-0461-3 Online measurement of cIPTG ester intermediates and NP-uncaging product. This figure shows the full data set of the measurement presented in Fig. 4 where measurements for 8, 15, 40 and 50 s of UV-A irradiation were not shown to increase readability. Fluorescence intensity (λ_Ex_ = 326 nm, λ_Em_ = 407 nm, black cross in Fig. 2) of 12 *E. coli* cultures before and after UV-A irradiation for 0–60 s (A) and fluorescence intensity measured directly after irradiation as a function of duration of UV-A exposure (B). At the beginning of the cultivation, 400 μM cIPTG were added to the medium. After 10 h, optical induction was performed with the LED array (λ_max_ = 368 nm, I = 52 mW/cm²). The amount of ester intermediates increases with increasing duration of UV-A exposure and can be fitted with first-order kinetics (solid lines and equations in B, R² > 0.995). Reduced irradiance leads to lower rate constants (black triangles, I = 13 mW/cm²) and reduced cIPTG concentration to lower amplitude (green diamonds, 50 μM cIPTG). Cultivation conditions: 800 μL Wilms-MOPS mineral medium (20 g/L glucose, 0.2 M MOPS) per well in a 48-FlowerPlate, 30 °C, shaking frequency: 1000 rpm, shaking diameter: 3 mm
10.1186/s12934-016-0461-3 Online measurement data for conventional induction profiling with manual addition of IPTG solution and optical induction profiling with cIPTG. This figure shows the full data set of induction profiling experiment presented in Fig. 6. Scattered light and FbFP fluorescence of 304 E. coli cultures induced with IPTG (A-F) and of 96 E. coli cultures induced with cIPTG (G-L). Time of induction and inducer strength (IPTG concentration or duration of UV-A exposure) are varied in full factorial design. Colors from blue to red mark later induction times (0.5–16 h), dull to bright colors mark increasing inducer strength (0–1000 μM IPTG or 0–40 s duration of UV-A exposure). The first column (A,D,G,J) shows the full data set while the second column (B,E,H,K) shows a subset at a fixed induction time of 7.5 h and the third column (C,F,I,L) shows a subset at a fixed inducer strength of 400 μM IPTG or 40 s UV-A exposure. Small colored down-pointing arrows illustrate the time of induction (not all shown). Long horizontal arrows in black illustrate general trends, e.g. impact of increasing inducer concentration on growth (B). Cultivation conditions: 800 μL Wilms-MOPS mineral medium per well in a 48-FlowerPlate, 400 μM cIPTG added to cultures induced with the LED array (λ_max_ = 368 nm, I = 52 mW/cm²), 30 °C, shaking frequency: 1000 rpm, shaking diameter: 3 mm
