# Supplementary figures and images for: Light-induced gene expression with photocaged IPTG for induction profiling in a high-throughput screening system
Source: Microb Cell Fact. 2016 Apr 23;15:63. doi: 10.1186/s12934-016-0461-3 (PMC4842301; doi:10.1186/s12934-016-0461-3)

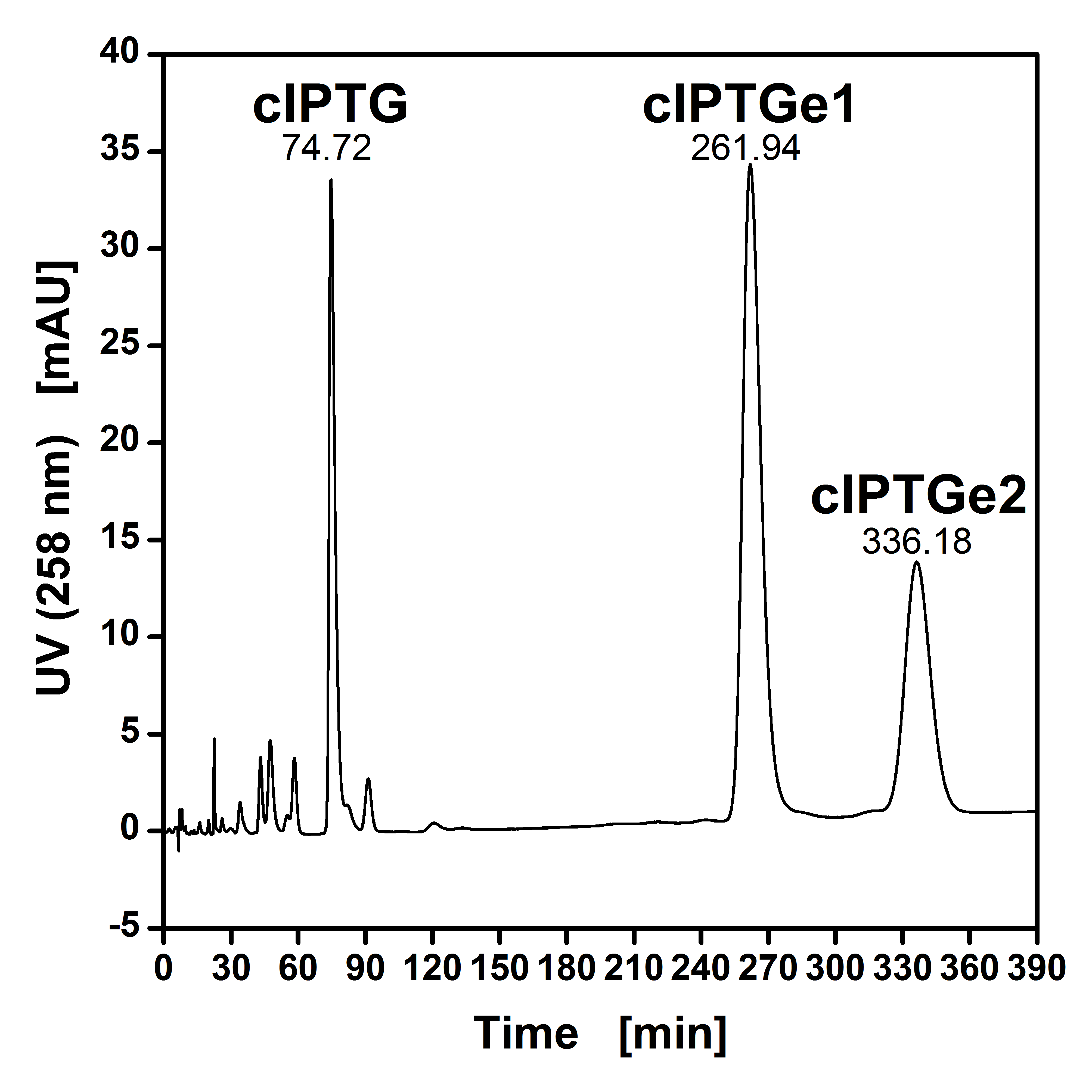

Supplement: Supplementary file 1 — 10.1186/s12934-016-0461-3 cIPTG was dissolved in isopropanol/n-heptan 50/50. (8.3 mg in 2.5 mL) and irradiated for 10 min (375 nm; 6.2 mW/cm²). cIPTG and its ester intermediates (cIPTGe1 and cIPTGe2) were then separated via HPLC (column: Chiralpak IC, 250·10 mm, Daicel, Japan; solvent: n-heptan:2-propanol (30:70); flow rate: 0.5 mL/min; detection: UV 258 nm) [file 12934_2016_461_MOESM1_ESM.tif]

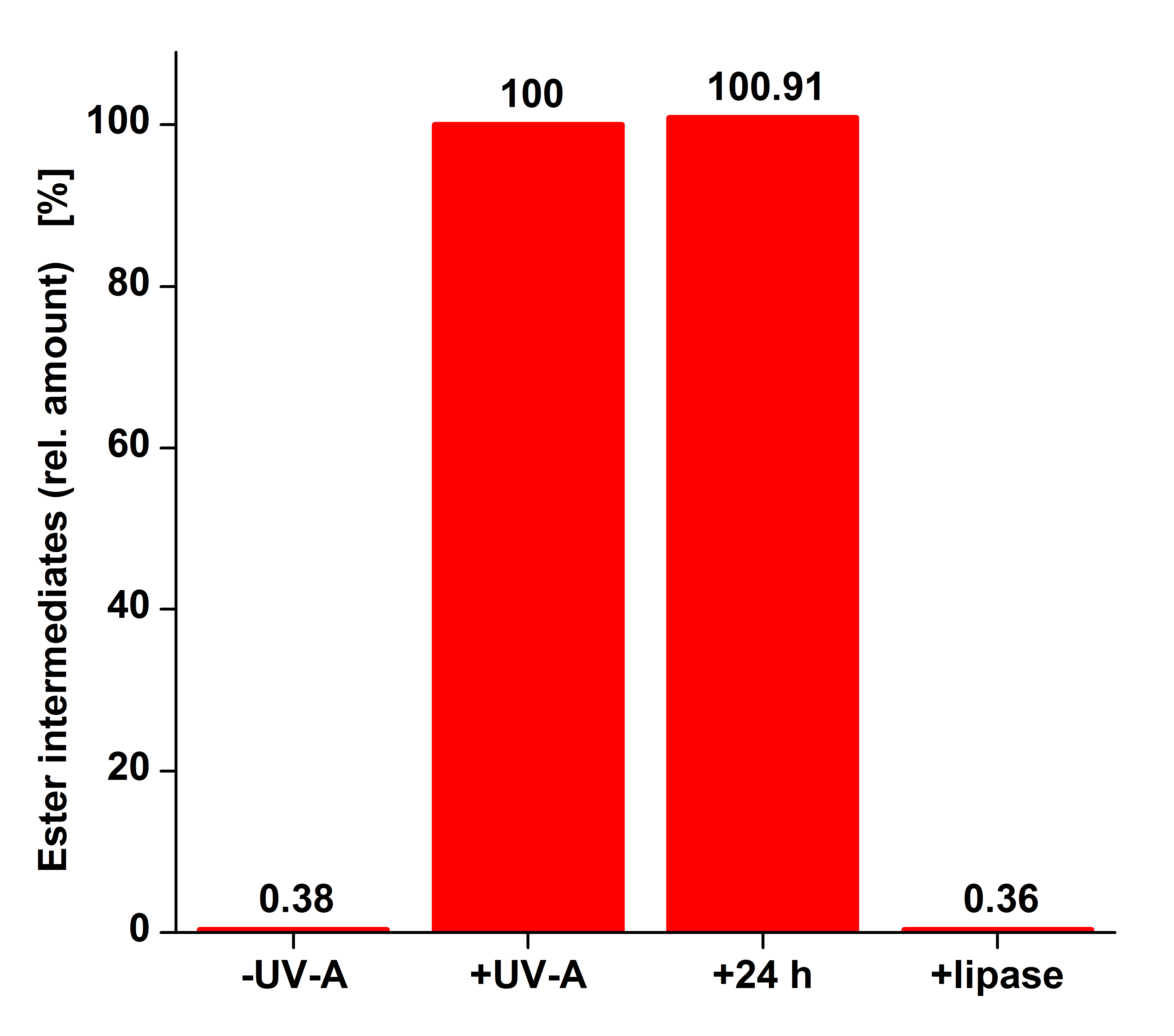

Supplement: Supplementary file 3 — 10.1186/s12934-016-0461-3 Relative amount of cIPTG ester intermediates over time. No ester intermediates are detected without UV-A irradiation (-UV-A). After UV-A irradiation (+UV-A) ester intermediates are detected. They were stable for at least 24 h (+24 h). Addition of lipase PL from Alcaligenes sp. fully degrades the ester intermediates (+lipase). HPLC (Jasco HPLC system, column: Hyperclone 5 μ ODS (C18) 120 (Phenomenex), solvent: MeOH:H2O 30:70, flow rate: 1 mL/min, 25 °C, 30 μL, detection: UV 258 nm at 11.46 min). 1000 μM cIPTG in H2O, irradiation with 6.4 mW/cm² at 375 nm for 10 min and storage at RT for 24 h, addition of 1 mg lipase PL (Alcaligenes sp. lipase 100000 U/g) to 910 μL at 38 °C for 24 h [file 12934_2016_461_MOESM3_ESM.tif]

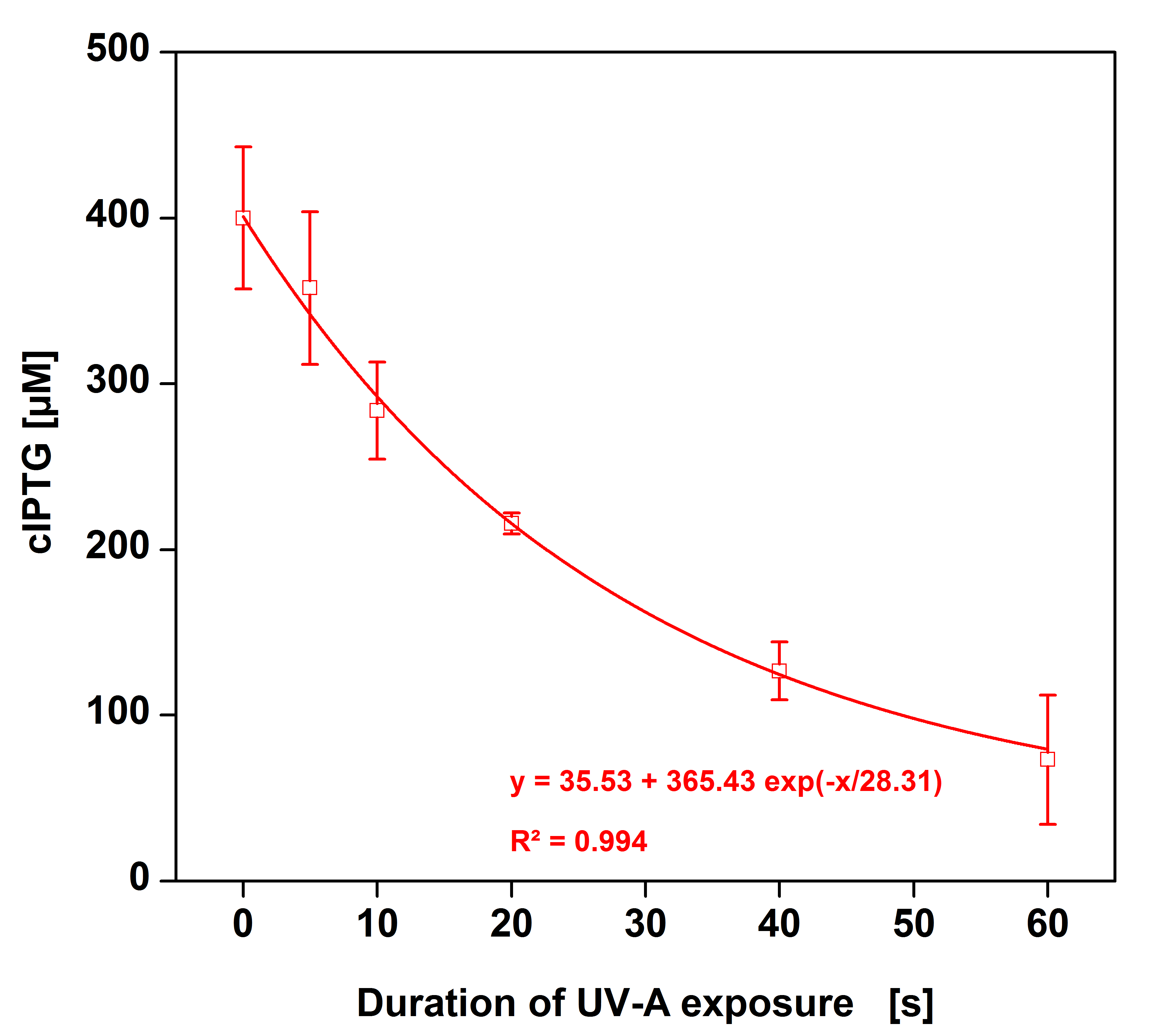

Supplement: Supplementary file 4 — 10.1186/s12934-016-0461-3 Photo-uncaging of cIPTG as a function of UV-A exposure duration. In vitro decomposition of 400 μM cIPTG in H2O by UV-A irradiation (λmax = 368 nm, I = 52 mW/cm², n = 4) monitored via HPLC–UV. HPLC (Jasco HPLC system, column: Hyperclone 5 μ ODS (C18) 120 (Phenomenex), solvent: MeOH:H2O 30:70, flow rate: 1 mL/min, 25 °C, 30 μL, detection: UV 258 nm at 19.04 min) [file 12934_2016_461_MOESM4_ESM.tif]

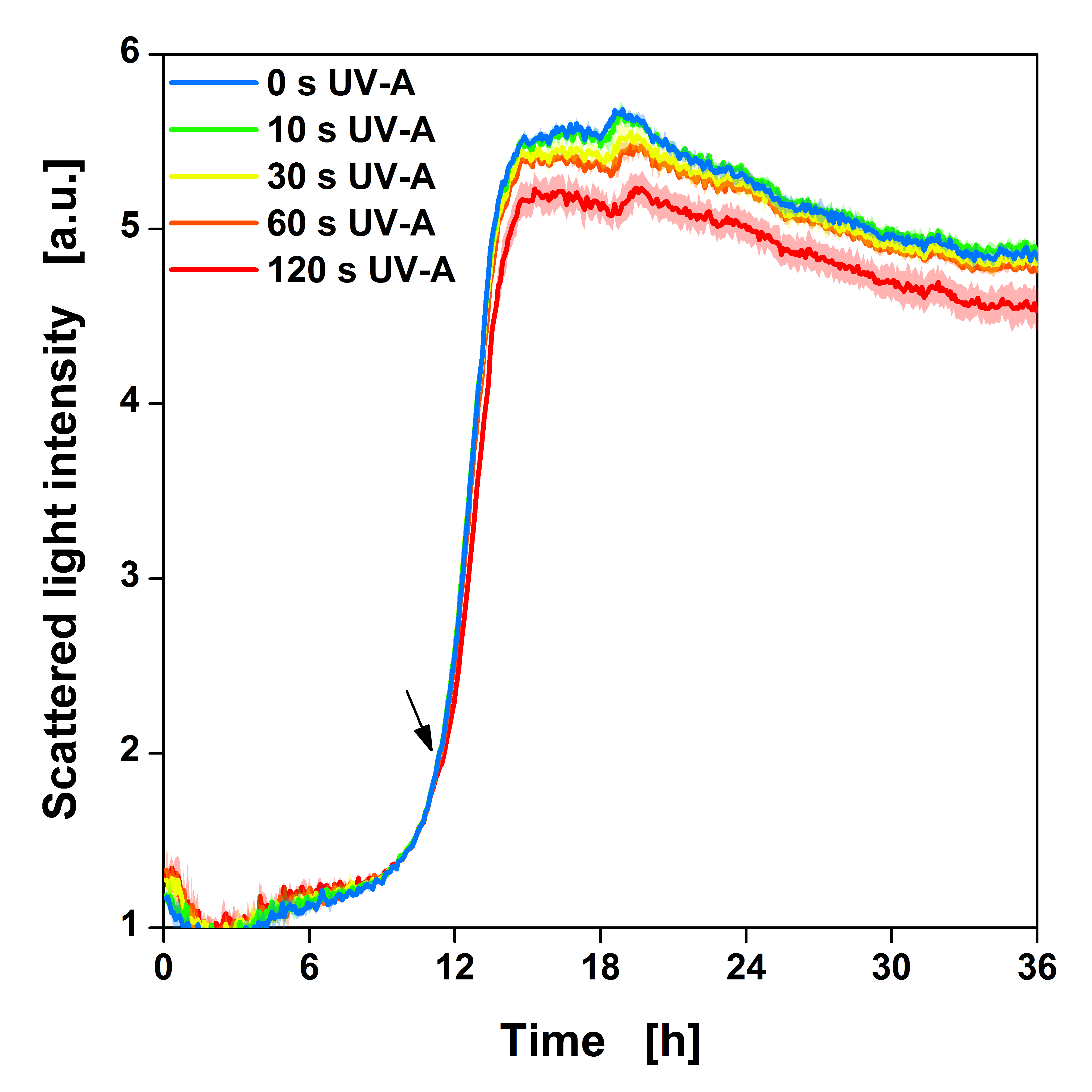

Supplement: Supplementary file 5 — 10.1186/s12934-016-0461-3 Effect of UV-A irradiation on cell growth. Scattered light intensity of non-induced cultures irradiated with UV-A LEDs for 0–120 s (λmax = 368 nm, I = 52 mW/cm²). No cIPTG was added to the medium. The black arrow indicates the time of UV-A exposure in the exponential phase. For up to 60 s of UV-A exposure only minute deviations are detected in the scattered light signal. Exposure for 120 s leads to a slightly lower scattered light signal in the stationary phase. Since exposure durations of up to 40 s were sufficient for optical induction, negative effects of UV-A irradiation are of no concern for the bacteria used in this work. Cultivations were performed in triplicates; standard deviation is shown in the same color as the mean value but at 50 % transparency [file 12934_2016_461_MOESM5_ESM.tif]

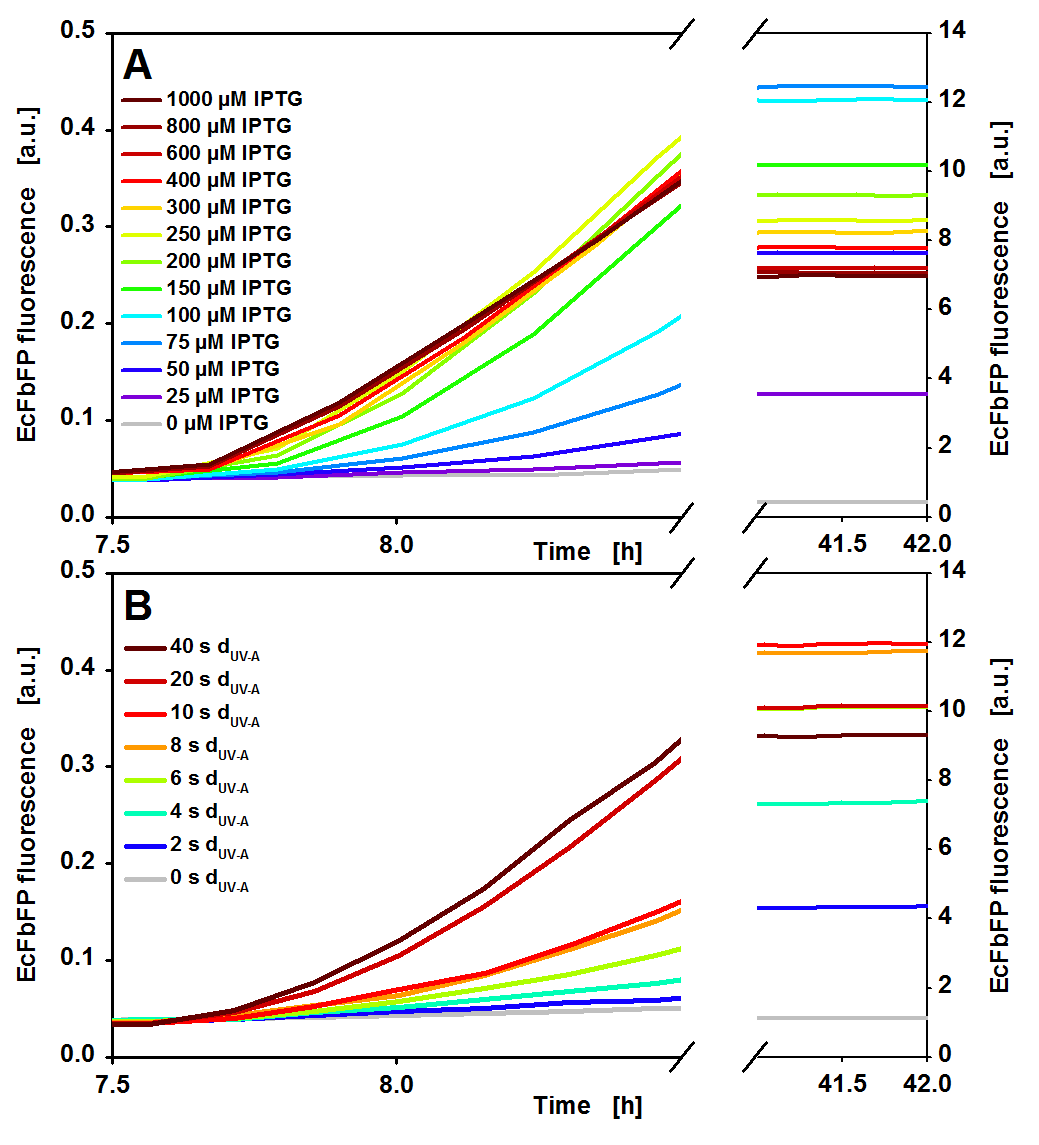

Supplement: Supplementary file 6 — 10.1186/s12934-016-0461-3 Initial product formation after induction. Zoomed view of Fig. 6c and Fig. 6g. FbFP fluorescence of E. coli cultures induced after 7.5 h with 0–1000 μM IPTG (A) or 400 μM of cIPTG and 0–40 s of UV-A irradiation (B). The initial product formation gradually increases with increasing IPTG concentrations (0–400 μM) and is saturated for higher concentrations (400–1000 μM) (A). However, the highest product fluorescence at the end of the cultivation after 42 h is reached with 75–100 μM IPTG (A, right side). For optical induction, initial product formation rate is highest for 20–40 s of UV-A irradiation and the highest product concentrations after 42 h are reached with 8–10 s (B). Note the axis scaling and breaks for increased readability. Additionally, note that only 400 μM of cIPTG are available for uncaging in B. Cultivation conditions: 800 μL Wilms-MOPS mineral medium per well in a 48-FlowerPlate, 400 μM cIPTG added to cultures induced with the LED array (λmax = 368 nm, I = 52 mW/cm²), 30 °C, shaking frequency: 1000 rpm, shaking diameter: 3 mm [file 12934_2016_461_MOESM6_ESM.tif]

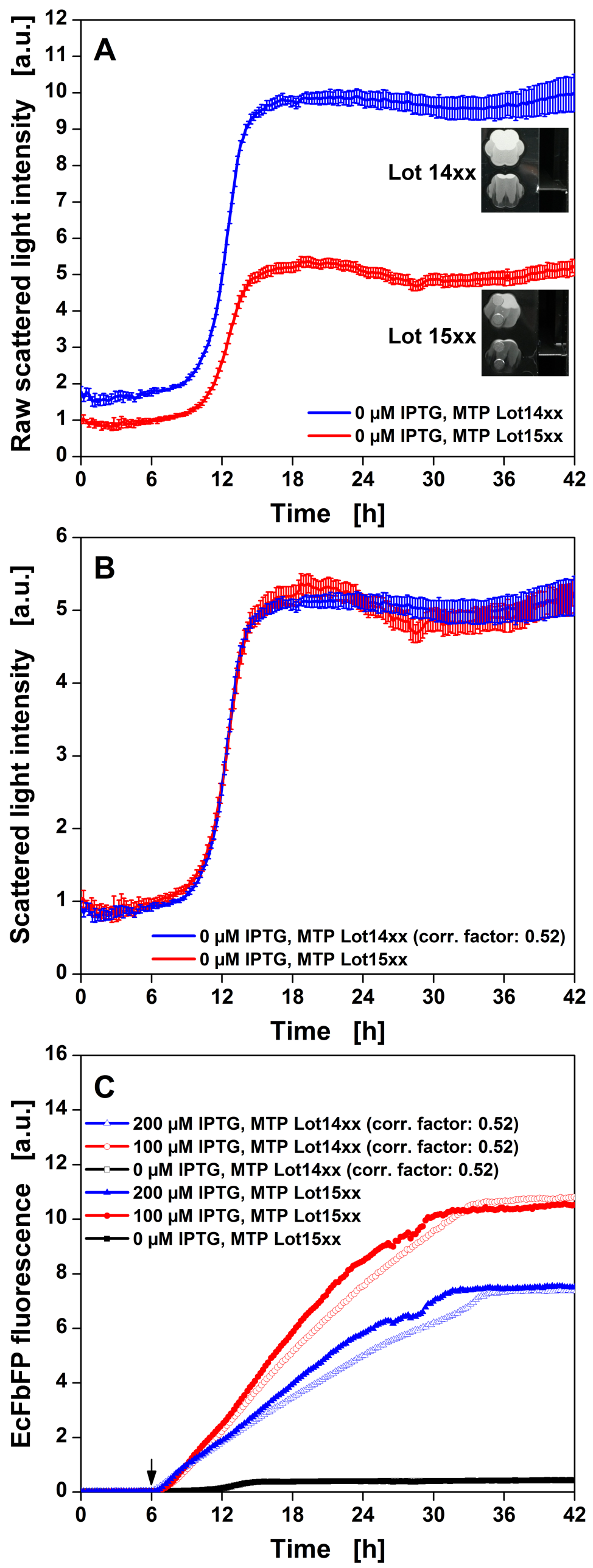

Supplement: Supplementary file 7 — 10.1186/s12934-016-0461-3 Data normalization. Raw scattered light signals are influenced by the microtiter plate lot (A). For normalization the raw signals of cultivations in one lot can be multiplied with a correction factor to match the course of the other cultivation (B). The correction factor is determined by dividing the scattered light intensities at the end of the cultivation after 42 h. This correction factor can also be applied to correct EcFbFP fluorescence signals (C). The normalized signals of cultures induced with 0, 100 or 200 μM IPTG are in good agreement. This demonstrates that reproducible results can be obtained even when different microtiter plate lots are used. Cultivation conditions: 800 μL Wilms-MOPS mineral medium per well in a 48-FlowerPlate, 30 °C, shaking frequency: 1000 rpm, shaking diameter: 3 mm. Error bars in A and B indicate the standard deviation of six reference cultures. Induction in C after 6 h. Data for 0 μM IPTG and lot 14xx is not visible in C because it is almost identical to lot 15xx [file 12934_2016_461_MOESM7_ESM.tif]

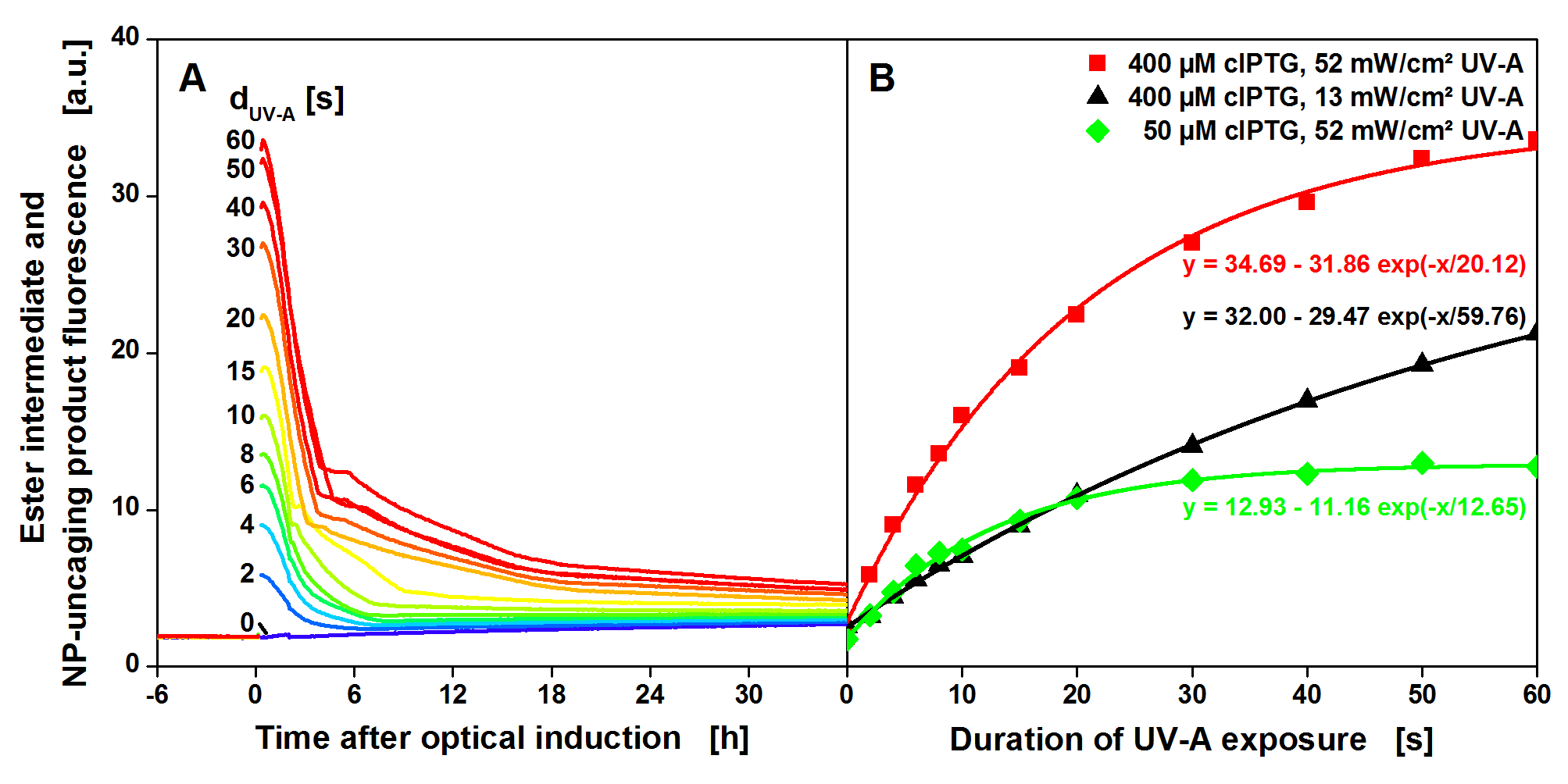

Supplement: Supplementary file 8 — 10.1186/s12934-016-0461-3 Online measurement of cIPTG ester intermediates and NP-uncaging product. This figure shows the full data set of the measurement presented in Fig. 4 where measurements for 8, 15, 40 and 50 s of UV-A irradiation were not shown to increase readability. Fluorescence intensity (λEx = 326 nm, λEm = 407 nm, black cross in Fig. 2) of 12 E. coli cultures before and after UV-A irradiation for 0–60 s (A) and fluorescence intensity measured directly after irradiation as a function of duration of UV-A exposure (B). At the beginning of the cultivation, 400 μM cIPTG were added to the medium. After 10 h, optical induction was performed with the LED array (λmax = 368 nm, I = 52 mW/cm²). The amount of ester intermediates increases with increasing duration of UV-A exposure and can be fitted with first-order kinetics (solid lines and equations in B, R² > 0.995). Reduced irradiance leads to lower rate constants (black triangles, I = 13 mW/cm²) and reduced cIPTG concentration to lower amplitude (green diamonds, 50 μM cIPTG). Cultivation conditions: 800 μL Wilms-MOPS mineral medium (20 g/L glucose, 0.2 M MOPS) per well in a 48-FlowerPlate, 30 °C, shaking frequency: 1000 rpm, shaking diameter: 3 mm [file 12934_2016_461_MOESM8_ESM.tif]

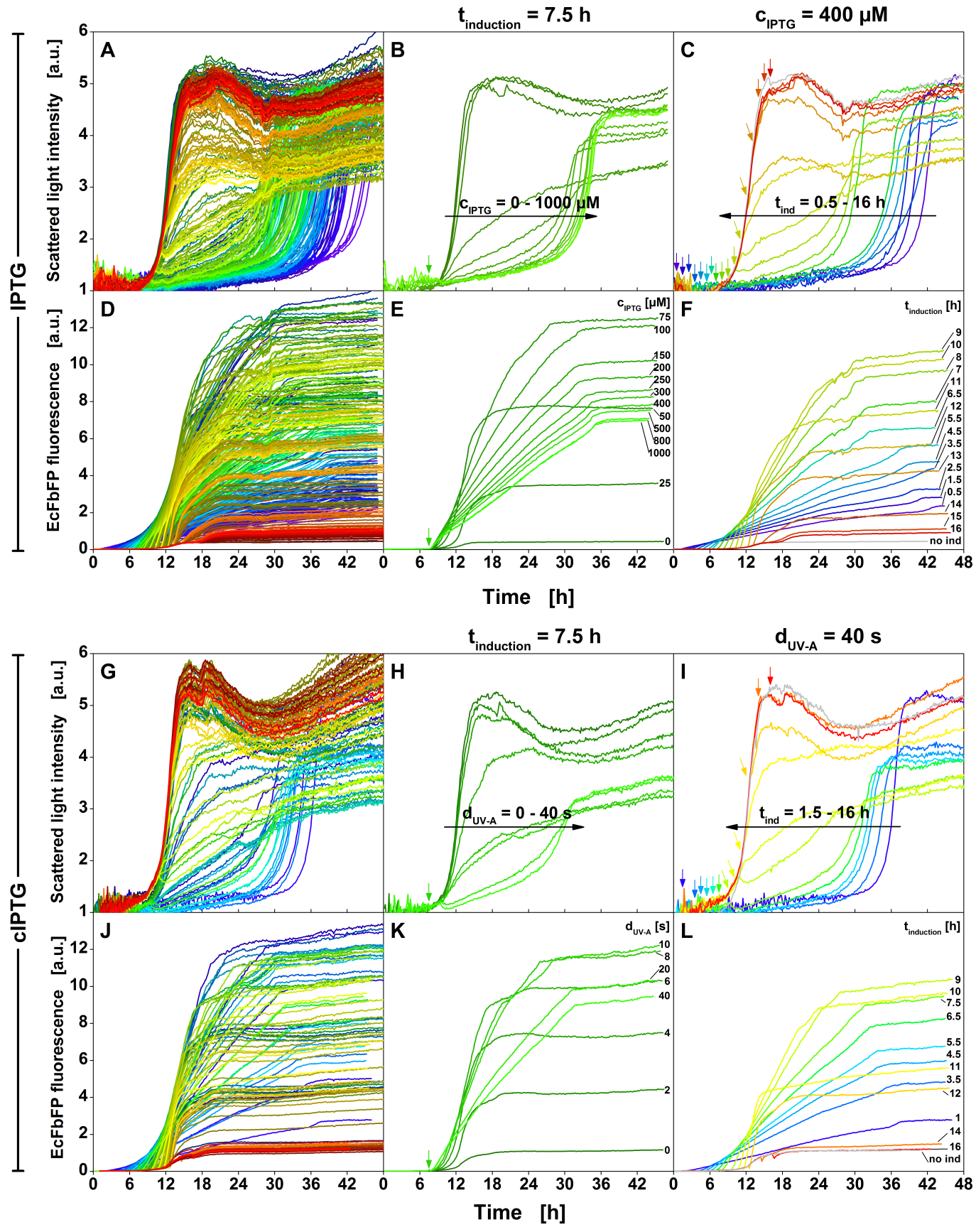

Supplement: Supplementary file 9 — 10.1186/s12934-016-0461-3 Online measurement data for conventional induction profiling with manual addition of IPTG solution and optical induction profiling with cIPTG. This figure shows the full data set of induction profiling experiment presented in Fig. 6. Scattered light and FbFP fluorescence of 304 E. coli cultures induced with IPTG (A-F) and of 96 E. coli cultures induced with cIPTG (G-L). Time of induction and inducer strength (IPTG concentration or duration of UV-A exposure) are varied in full factorial design. Colors from blue to red mark later induction times (0.5–16 h), dull to bright colors mark increasing inducer strength (0–1000 μM IPTG or 0–40 s duration of UV-A exposure). The first column (A,D,G,J) shows the full data set while the second column (B,E,H,K) shows a subset at a fixed induction time of 7.5 h and the third column (C,F,I,L) shows a subset at a fixed inducer strength of 400 μM IPTG or 40 s UV-A exposure. Small colored down-pointing arrows illustrate the time of induction (not all shown). Long horizontal arrows in black illustrate general trends, e.g. impact of increasing inducer concentration on growth (B). Cultivation conditions: 800 μL Wilms-MOPS mineral medium per well in a 48-FlowerPlate, 400 μM cIPTG added to cultures induced with the LED array (λmax = 368 nm, I = 52 mW/cm²), 30 °C, shaking frequency: 1000 rpm, shaking diameter: 3 mm [file 12934_2016_461_MOESM9_ESM.tif]
